# Dissociation of Circadian and Circatidal Timekeeping in the Marine Crustacean *Eurydice pulchra*

**DOI:** 10.1016/j.cub.2013.08.038

**Published:** 2013-10-07

**Authors:** Lin Zhang, Michael H. Hastings, Edward W. Green, Eran Tauber, Martin Sladek, Simon G. Webster, Charalambos P. Kyriacou, David C. Wilcockson

**Affiliations:** 1 Department of Genetics, University of Leicester, Leicester LE1 7RH, UK; 2 MRC Laboratory of Molecular Biology, Cambridge CB2 0QH, UK; 3 School of Biological Sciences, Bangor University, Bangor LL57 2UW, UK; 4 Institute of Biological, Environmental, and Rural Sciences, Aberystwyth University, Aberystwyth SY23 3DA, UK

## Abstract

**Background:**

Tidal (12.4 hr) cycles of behavior and physiology adapt intertidal organisms to temporally complex coastal environments, yet their underlying mechanism is unknown. However, the very existence of an independent “circatidal” clock has been disputed, and it has been argued that tidal rhythms arise as a submultiple of a circadian clock, operating in dual oscillators whose outputs are held in antiphase i.e., ∼12.4 hr apart.

**Results:**

We demonstrate that the intertidal crustacean *Eurydice pulchra* (Leach) exhibits robust tidal cycles of swimming in parallel to circadian (24 hr) rhythms in behavioral, physiological and molecular phenotypes. Importantly, ∼12.4 hr cycles of swimming are sustained in constant conditions, they can be entrained by suitable stimuli, and they are temperature compensated, thereby meeting the three criteria that define a biological clock. Unexpectedly, tidal rhythms (like circadian rhythms) are sensitive to pharmacological inhibition of Casein kinase 1, suggesting the possibility of shared clock substrates. However, cloning the canonical circadian genes of *E. pulchra* to provide molecular markers of circadian timing and also reagents to disrupt it by RNAi revealed that environmental and molecular manipulations that confound circadian timing do not affect tidal timing. Thus, competent circadian timing is neither an inevitable nor necessary element of tidal timekeeping.

**Conclusions:**

We demonstrate that tidal rhythms are driven by a dedicated circatidal pacemaker that is distinct from the circadian system of *E. pulchra*, thereby resolving a long-standing debate regarding the nature of the circatidal mechanism.

## Highlights

• The intertidal crustacean *Eurydice pulchra* exhibits circadian and tidal phenotypes
• We have cloned and characterized the canonical circadian factors of *Eurydice*
• While sensitive to CK1 inhibition, circadian and tidal clocks can be dissociated
• *Eurydice* has a dedicated circatidal clock independent of its circadian clock

## Graphical Abstract

**Figure:**
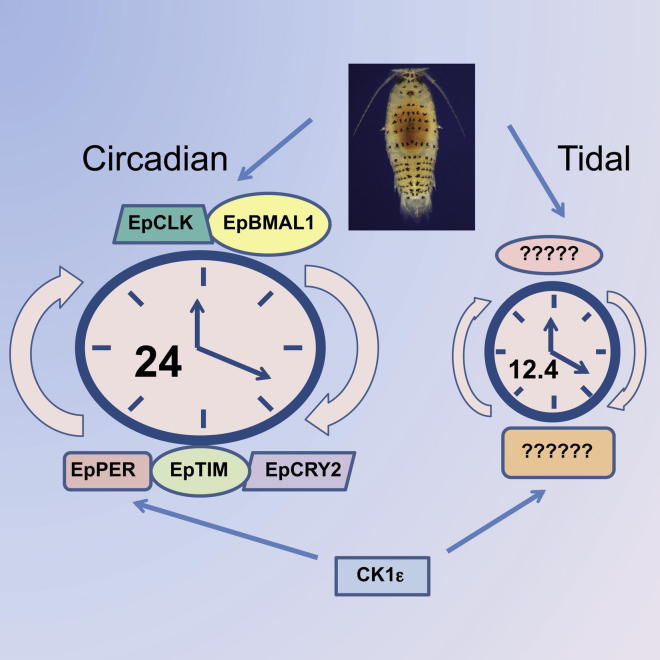

## Introduction

Circadian timekeeping, driven by intrinsic clocks with a period of approximately 24 hr, is common to all kingdoms of life. Its adaptive value is that it allows an organism to anticipate the regular environmental changes associated with the solar and seasonal cycles of day and night. The molecular mechanisms of circadian clocks, revealed in model organisms from Cyanobacteria to mammals, pivot around negative and positive transcriptional feedback loops allied to posttranscriptional and posttranslational processes that together generate a biological cycle of approximately 24 hr [1–3].

Species inhabiting coastal environments are, however, challenged with considerably more complex temporal patterns, dominated by tidal and lunar cycles [4]. Consequently, intertidal plants and animals show adaptive, free-running ∼12.4 hr (i.e., circatidal) rhythms of behavior, metabolism, and reproduction that are synchronized to the tidal environment by relevant cues, including turbulence/vibration, moonlight, salinity, and temperature changes [5]. Such free-running rhythms suggest the presence of endogenous circatidal clocks, but despite extensive behavioral descriptions, the molecular components of tidal clocks are largely unexplored. Indeed, their independent existence is questioned by the view that the tidal mechanism is simply a submultiple of the 24 hr clock, sharing circadian components to generate two oscillators whose outputs are in antiphase ∼12.4 hr apart [6]. The alternative view is of a dedicated, independent circatidal (12.4 hr) oscillator that may interact with the circadian clock but that uses different molecular components [5]. This debate has persisted for many years in the absence of any definitive experiments.

To address the issue of molecular independence directly, we revisited decades-old studies on *Eurydice pulchra* (Leach), an intertidal isopod crustacean that exhibits robust tidal behavior [7, 8]. Our rationale was to exploit environmental, pharmacological and genetic means to test the interdependence of circadian and circatidal timekeeping. Critical to this approach was the cloning of the canonical circadian clock genes of *Eurydice*, because this would provide molecular markers for the circadian mechanism and also potential targets with which to disrupt it. We could then test the expression of tidal phenotypes in the absence of circadian competence. Sustained circatidal timekeeping under such circumstances would demonstrate its separate identity and distinct molecular machinery, thereby resolving a long-standing problem in chronobiology.

## Results

### Tidal and Circadian Elements of Behavior and Physiology in *Eurydice*

**Figure 1:**
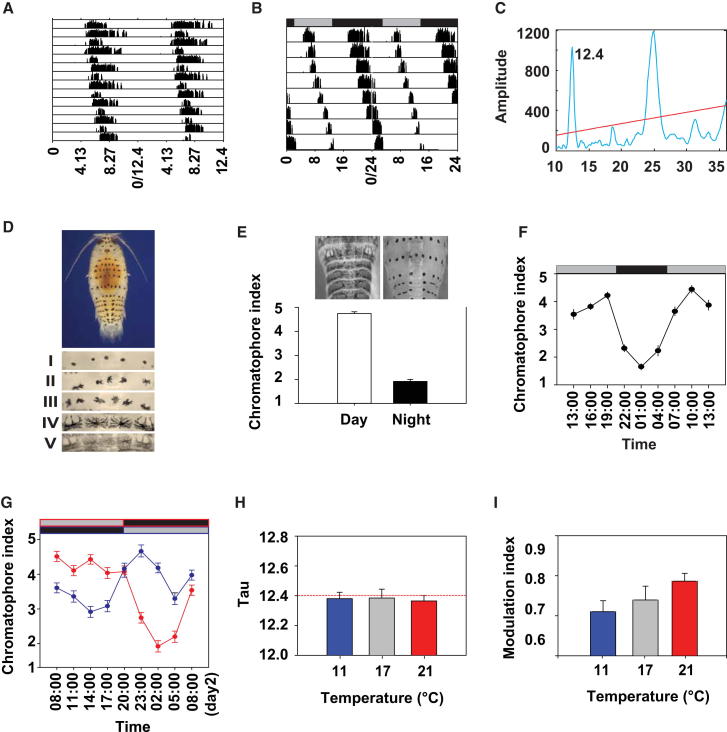
Tidal and Circadian Control of Behavior and Physiology in *Eurydice* (A) Shore-caught *Eurydice* show robust circatidal swimming in DD. An individual actogram, double plotted on 12.4 hr time base over 7 days, is shown. (B) The same data as in (A) double-plotted on a 24 hr time base to show more clearly the daily modulation of swimming episodes. (C) Periodogram for the animal in (A) and (B). Red line, p < 0.001 level. (D) Dorsal chromatophores of *Eurydice* and respective pigment dispersion index scale I to V. (E) Chromatophores of animals from the beach show pigment dispersion during the day (mean + SEM, F_1,145_ = 2.13, p = 0.003). (F) Chromatophore pigment dispersion (mean + SEM) in *Eurydice* removed from the shore and released into DD. Gray/black bars show expected light regime on the home beach (see also Figure S1). (G) Chromatophore pigment dispersion (mean + SEM) in *Eurydice* entrained in reversed LD 12:12 and released into DD. (H) The tidal clock is temperature compensated. The period of swimming rhythms in beach-caught animals free running at 11°C, 17°C (ambient seawater temperature) and 21°C is shown. The red dotted line indicates a 12.4 hr period (mean + SEM, n = 32–58). (I) The daily modulation of tidal activity is temperature compensated (MI data mean + SEM, n = 32–58). See also Figure S1.

*Eurydice* individuals taken from the shore and placed immediately into constant darkness (DD) exhibited clear and sustained circatidal swimming rhythms (Figures 1A–1C). Of 48 animals tested, 40 (83%) gave a statistically significant tidal cycle with a period of 12.43 + 0.03 hr (mean + SEM). The remainder were arrhythmic or exhibited very low levels of activity. Importantly, the activity pattern was amplitude modulated, the levels of swimming during subjective night (SN) being considerably higher than during the subjective day (SD) (Figures 1A and 1B). This was represented by a “modulation index” (MI), calculated as the total activity during SN as a proportion of total SD + SN activity. Whereas 0.50 reflects the absence of modulation, animals free running in DD after beach collection exhibited an MI of 0.77 + 0.04.

Animals from the beach exhibited a second daily phenotype, the cycle of pigment dispersion in the chromatophores [9], which was high during daytime (Figures 1D and 1E) and reduced at night. This rhythm is circadian as it persisted in animals transferred to DD in the laboratory (Figure 1F) with a significant peak during subjective day (F_8,284_ = 42.6, p ≈ 0). To study entrainment of this rhythm by light, we held animals in DD for 2 weeks, then exposed them to a 12:12 light-dark (LD) cycle for 5 days and then returned them to DD and sampled them over days 2 and 3. This revealed a significant circadian rhythm of chromatophore dispersal (F_15,376_ = 7.29, p ≈ 0), with higher dispersal during the subjective day, in phase with the preceding LD cycle and with a period of 25.4 hr (cosinor F_3,12_ = 18.48, p = 0.0003; Figure S1 available online). Finally, to confirm entrainment by light, we entrained the chromatophore rhythms to reverse LD 12:12 cycles and observed that the corresponding chromatophore cycles were in antiphase to each other and shifted by ∼12 hr (phase × time interaction F_8,266_ = 88.5, p ≈ 0; Figure 1G).

A defining feature of biological clocks is temperature compensation: the ability to maintain constant period over a wide temperature range [10]. We therefore examined free-running tidal periodicity of animals taken from the beach and placed immediately in DD and held between 11°C and 21°C for 5 days. In the absence of compensation, the period at 11°C should be twice as long as that at 21°C. The tidal period measured was, however, constant over this temperature range (Figure 1H; F_2,140_ = 0.35, p = 0.71). Moreover, amplitude modulation of tidal behavior was robust under all three temperatures (MI = 0.71 to 0.78, F_2,108_ = 2.62, p = 0.077; Figure 1I). This confirmed that the mechanism responsible for the timing of the modulation is temperature compensated because if it were not, it would have lost temporal coincidence with the tidal peaks, and thus daily modulation would have been compromised. Thus, the properties of the overt tidal and circadian phenotypes reveal bona fide underlying clock mechanisms.

### Pharmacological Manipulation of Tidal and Circadian Behavior and Physiology in *Eurydice*

**Figure 2:**
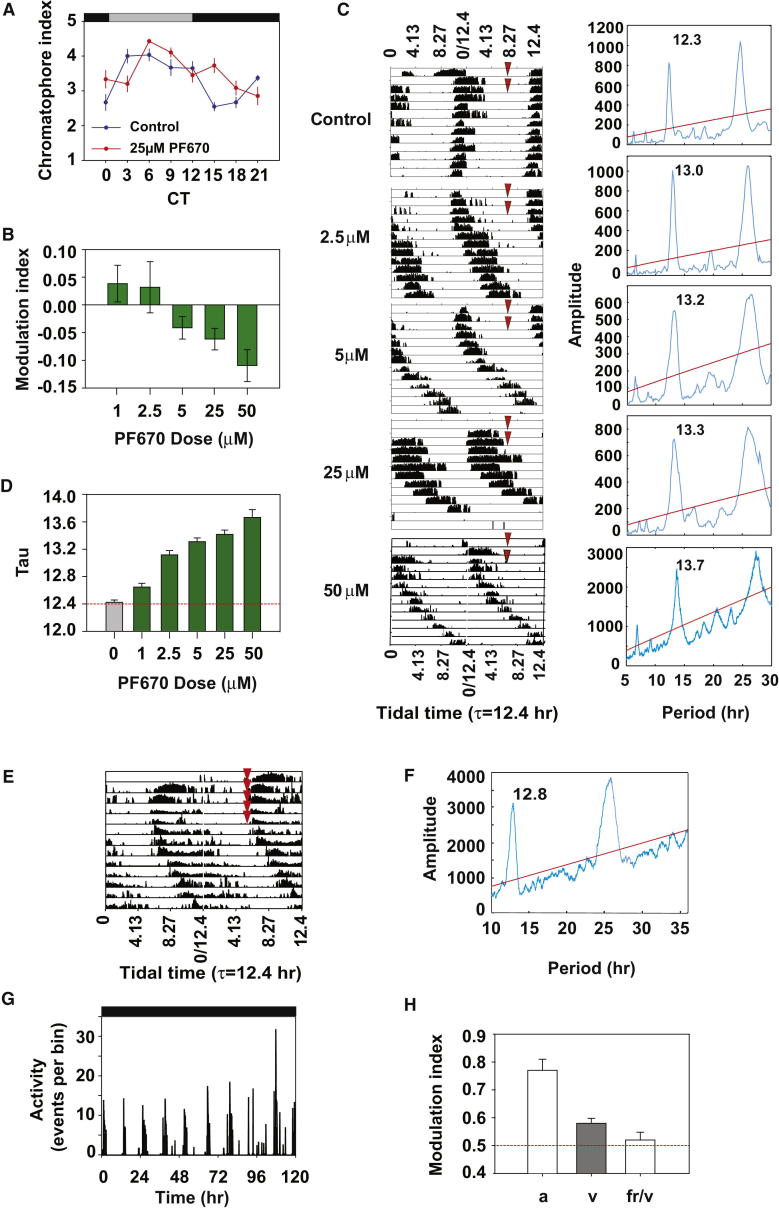
Manipulation of Tidal and Circadian Behavior and Physiology in *Eurydice* by Casein Kinase Inhibitor and Periodic Vibration (A) Chromatophore index (mean ± SEM) for animals in DD exposed to 25 μM PF670462 (red) or vehicle (blue). (B) Dose-response curve for daily modulation of tidal behavior (MI, mean + SEM) by PF670462. (C) Free-running actograms (left) for individuals administered different doses of PF670462 (red arrows) and their corresponding periodograms (right). (D) Free-running period of tidal activity rhythm shows a dose-response relationship for PF670462 (mean + SEM; see Figure S2 for PF4800567 results). (E) Representative activity trace shows entrainment of previously arrhythmic *Eurydice* tidal behavior by periodic vibration in DD (red arrows) after release to free run. (F) Periodogram reveals a 12.8 hr period in DD after vibration. (G) Representative activity trace shows that entrainment of tidal behavior by vibration does not restore amplitude modulation of swimming. (H) Modulation indices (mean + SEM) of groups of animals in DD taken from the beach (a, n = 14), during vibration entrainment (v, n = 15) and in free run after vibration (fr/v, n = 14). See also Figure S2.

In contrast to the period-stabilizing effects of temperature compensation, various small molecules have been shown to affect circadian rhythms [11–13], but pharmacological manipulation of tidal time keeping is unexplored. In an attempt to differentiate the putative circadian and tidal clocks pharmacologically, we screened compounds previously shown to affect circadian period in mammalian tissues for their effects on circadian timing in *Eurydice*. We anticipated circadian timing to be sensitive to such manipulation, but that circatidal timing would be insensitive, if the two oscillators are independent. PF670462 is an inhibitor of both mCK1δ/ε isoforms and blocks the phosphorylation and degradation of mPER, thereby lengthening circadian period in mammals [11]. Consistent with an anticipated circadian effect, PF670462 (25 μM in seawater) significantly damped the chromatophore rhythm (Figure 2A; ANOVA, treatment F_1,437_ = 3.89, p = 0.049, time F_7,437_ = 10.7, p ≈ 0, time × treatment F_7,437_ = 5.65, p = 3 × 10^−6^). The reduced amplitude of the PF670462 cycle was also associated with a phase delay suggestive of a longer period. The drug also suppressed the daily modulation of tidal behavior dose dependently, when compared to simultaneously run nondrugged controls (F_4,159_ = 4.26, p = 0.003; Figure 2B). Surprisingly, however, tidal periodicity was also dramatically and dose-dependently lengthened (F_5,187_ = 57.1, p ≈ 0; Figures 2C and 2D) by CK1 inhibition. The tidal cycles remained well defined throughout the recordings, and activity levels were similar at all doses, suggesting that the animals were not behaviorally compromised. Thus, PF670462 appears to affect both circadian and tidal rhythms in a way that is reminiscent of its effects on mammalian circadian rhythms [11], suggesting the possibility that the mechanisms underlying the two types of rhythms are intimately related or share a common involvement of CK1. Furthermore, the tidal effect was specific for CK1 because a second compound, PF-4800567 (12.5 and 25 μM), which is more specific for mCK1ε [11], also similarly lengthened circatidal period (12.5 μM, period = 12.65 hr ± 0.14; 25 μM, period = 13.17 hr ± 0.06 SEM; Figure S2). Consequently, although we were unable to differentiate circadian and tidal mechanisms by this pharmacological approach, we were able to reveal an unexpected pharmacological sensitivity of the putative tidal pacemaker. The stability and precision of the tidal rhythm even when period was extended pharmacologically by over 10% emphasizes further the robustness of the underlying pacemaking mechanism.

### Entrainment by Vibration Separates Tidal and Circadian Behavior in *Eurydice*

In a further attempt to separate tidal and circadian timing, we used tidal entrainment by vibration to restore rhythms in animals that had become arrhythmic following maintenance in DD for more than 1 month. A vibration stimulus was applied for 10 min every 12.4 hr for 5 cycles in DD before the animals were left to free run for 8 days (Figure 2E). Of 21 animals, 15 (71%) showed behavioral entrainment, with a significant tidal period of 12.7 + 0.15 hr, but showed little daily modulation (MI = 0.58 + 0.02; nota bene MI = 0.50 represents no modulation; Figures 2E–2H). All animals also gave a significant tidal period during the subsequent free run (12.84 + 0.18 hr), but with no significant daily modulation (MI = 0.52 + 0.03 hr; Figures 2G and 2H). There were no significant differences in period (F_1,28_ = 0.25, p = 0.62) or MI (F_1,28_ = 1.97, p = 0.21) between the entrained and free-run intervals.

Consequently, modulation of swimming episodes is lost after prolonged periods in DD, revealing that it is not an intrinsic property of the tidal clock but rather that it is likely driven by a circadian mechanism (see below) that is not an inevitable component of tidal behavior. Conversely, tidal rhythms can be entrained by vibration and expressed in the absence of any putative circadian modulation (Figures 2G and 2H). *Eurydice* thus demonstrates the three canonical features of a true tidal clock: free-running rhythmicity, temperature compensation, and entrainment by appropriate stimuli.

### Molecular Cloning and Expression of Canonical Circadian Clock Genes in *Eurydice*

To facilitate the distinction between tidal and circadian events, we attempted to extend our circadian phenotypes by examining circadian gene expression. We employed library screening and PCR amplification to clone and identify full-length sequences for *Eurydice pulchra period* (*Epper*), *timeless* (*Eptim*), *Clock* (*EpClk*), *bmal1* (*Epbmal1*), *cryptochrome2* (*Epcry2*), *6-4 photolyase* (Figure S3 and Table S1), and *Ck1* (*EpCK1ε*) alongside partial sequences for other clock-relevant kinases, phosphatases, and components of degradation pathways. We were unable to identify a *Drosophila*-like *cry* molecule or a *CK1δ* sequence. Comparison of domain structures of canonical clock proteins (Figure S3A) revealed that, like *Daphnia*, EpPER has a CK1 binding region with similarity to mouse PER1 and *Drosophila* PER [14, 15]. Moreover, *Eurydice* clusters with the vertebrate-like BMAL1 sequences that have the extended C-terminal containing a putative transactivation domain absent in *Drosophila* CYC (Figures S3A and S3B). Here, all CYC proteins with a C-terminal conserved transactivation domain were designated as BMAL1. *Eurydice* CRY is a vertebrate-like CRY2 sequence (Figures S3A and S3C) and EpTIM clusters with TIM rather than the paralog TIM2/TIMEOUT (Figure S3D). The presence of a vertebrate-like EpCRY2 (Figures S3A and S3C) suggests that it could represent a negative regulator for the circadian clock of *Eurydice*. Finally, of a number of splice isoforms, EpCLK5 was the most highly expressed in heads (Figure S3E).

**Figure 3:**
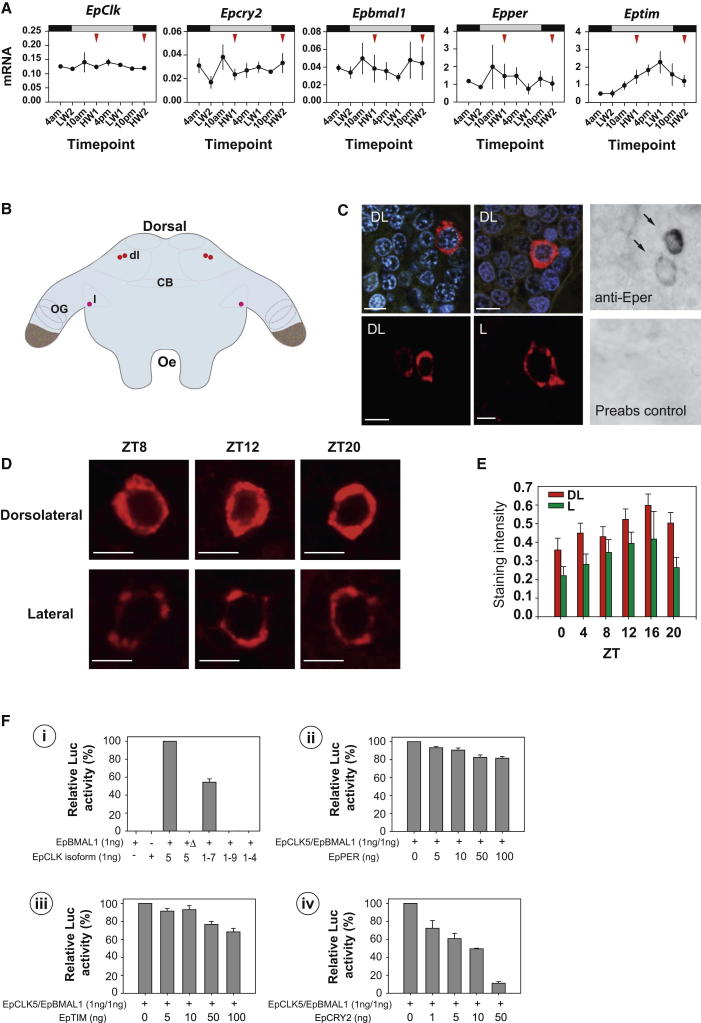
Characterization of Canonical *Eurydice* Circadian Clock Genes (A) Only *Eptim* in *Eurydice* heads shows circadian cycling in DD. Mean abundance (±SEM) in copy number per 100 copies of the reference gene *Eprpl32* is shown Horizontal bars, expected light and dark cycles; red arrowheads, time of expected high water; LW and HW, low and high water. LW2 is equivalent to CT0. (See Figures S3A–S3F and Table S1 for details of *Eurydice* clock genes.) (B) Cartoon of *Eurydice* brain illustrating the relative position of cells immunopositive to anti-EpPER sera. Red, dorsolateral (dl); pink, lateral (l); OG, optic ganglia; CB, central body; Oe, esophagus. (C) Anti EpPER immunoreactivity in the brain of *Eurydice*. The upper colored panels show dorsolateral cells at ZT15, counterstained with DAPI. The panel on the left shows strong cytoplasmic immunoreactivity. The right-hand panels show partial nuclear labeling in addition to cytoplasmic localization. The lower left shows paired dorsolateral (DL) cells taken from one hemisphere (ZT8). The lower right shows anti-EpPER-positive cell in the lateral (L) region (ZT8). Scale bars represent 15 μm. The black and white sections show that preabsorption of the EpPER antiserum with the cognate peptide (lower panel) eliminated immunostaining (arrows) of dorsolateral neurons (see also Figure S3G). (D) Anti-EpPER immunoreactivity of dorsolateral and lateral cells at different times of the day (ZT0, lights on; ZT12, lights off) showing predominantly cytoplasmic and partial nuclear labeling. Scale bars represent 10 μm. (E) EpPER antigenicity in dorsolateral and lateral cells under LD 12:12 cycles. (F) EpPER, EpTIM, and EpRCRY2 negatively regulate EpCLK-EpBMAL1-mediated transcription in *Drosophila* S2 cells. n = 3–6. (See also Figure S3H and Table S2 for EpPER functional analysis in transgenic flies.) (Fi) EpCLK isoforms 5 and 1-7 activate E-box mediated luciferase activity, whereas the other isoforms, 1-9 (missing part of PAS-B) and 1-4 (lost most of polyQ region), do not (see Figure S1E). Deletion of the putative BMAL1 C-terminal transactivation domain (+Δ) does not activate transcription. Mean Luc activity (+SEM) normalized to *Renilla* is shown. (Fii and Fiii) EpPER (Fii) and EpTIM (Fiii) modestly repress EpCLK-BMAL1 mediated activation. (Fiv) EpCRY2 robustly represses luciferase activity. See also Figure S3 and Tables S1 and S2.

*Epper*, *Eptim*, *Epbmal1*, *EpClk*, and *Epcry2* transcripts were expressed in several tissues, including brain, ventral nerve cord, gut, hepatopancreas, and ovary (Figure S3F). Only *Eptim*, however, gave robust and reliable circadian expression in the head, with a peak late in the subjective day in freshly collected, tidally active animals (Figure 3A). Importantly, there was no indication of any significant bimodal expression in any of these transcripts that might indicate an underlying tidal oscillation. Rhythmic expression of *Eptim*, comparable to the circadian cycle of *tim* seen in *Drosophila* [16], therefore provides a molecular marker for the circadian oscillator of *Eurydice*.

Attempts to raise antisera to EpTIM and EpCRY2 were unsuccessful, but we were able to raise a custom-made polyclonal antiserum against EpPER peptide (see Figures 3C and S3G for details on specificity). We therefore mapped the expression of EpPER in the *Eurydice* brain and identified immunoreactivity in a pair of cells located dorsolaterally in the brain (Figures 3B–3D). A further cell located laterally also expressed EpPER. All three cells revealed strong cytoplasmic but only weak nuclear expression (Figures 3C and 3D). We then examined the intensity of EpPER antigenicity every 4 hr in an LD 12:12 cycle. In both the dorsolateral and lateral cells we did not detect significant time differences even though there was a suggestion of a cycle peaking at night (F_5,126_ = 1.95, p = 0.092, and F_5, 41_ = 1.08, p = 0.38, respectively; Figure 3E). When we collapsed the dorsolateral cell data into daytime versus nighttime intensity, there was significantly higher EpPER intensity at night but not for the lateral cells (F_1,116_ = 4.7, p = 0.031, and F_1,45_ = 0.77, p = 0.39, respectively; Figure 3E). Finally, we compared the location of these putative circadian clock cells with the previously identified *Eurydice* PDH cells [17], a marker for a subset of circadian neurons in *D. melanogaster* [18]. It was clear that the EpPER-expressing cells were not the PDF-positive neurons.

### Transcriptional Regulatory Actions of *Eurydice* Circadian Clock Genes Revealed in *Drosophila* S2 Cells

To define their putative transcriptional actions, we expressed the *Eurydice* clock proteins in *Drosophila* S2 cells cotransfected with an *E-box-luciferase* (*E-box-luc*) reporter. S2 cells endogenously express dCYC, but addition of different EpCLK isoforms alone did not activate *E-box-luc* (Figure 3Fi). We therefore tested each EpCLK isoform with EpBMAL1, and we found that *EpClk5*, the most abundant head isoform (Figure S3E), was the most effective transactivator (p < 0.0002 compared to EpCLK1-7; Figure 3Fi). Deletion of the C-terminal domain of EpBMAL1 (EpBMAL1Δ) reduced *E-box-luc* expression to baseline levels, consistent with its proposed transactivation function (Figure 3Fi). Both EpPER and EpTIM transfected individually had a significant but modest repressive effect on EpCLK-EpBMAL1-mediated transcription (F_5,12_ = 14.99, p < 0.0001, and F_5,12_ = 8.2 p = 0.0014, respectively; Figures 3Fii and 3Fiii). The most dramatic repression was generated by EpCRY2 (F_4,10_ = 14.95, p = 0.0003; Figure 3Fiv), suggesting EpCRY2 as the major putative negative regulator in *Eurydice*’s circadian mechanism.

As a further analysis of circadian function, we tested whether any *Eurydice* clock factor might rescue circadian activity rhythms in null mutant *Drosophila. Epcry2* was not tested because *Drosophila* does not have an ortholog. We therefore generated a *UAS-Epper* transgene and transformed it into arrhythmic *per*^*01*^ *D. melanogaster* hosts. Two lines with randomly integrated inserts were crossed to the *tim-gal4* driver and locomotor behavior was monitored under DD at 25°C. Although modest when compared to the conspecific *D. melanogaster UAS-Dmper* transgene, both independent insertions partially rescued the *per*^*01*^ phenotype, with about one-third of flies showing significant periodicity in the circadian range (Figure S3H and Table S2). Hence, *Epper* shows some circadian competence within the *Drosophila* clock mechanism implying a similar function in *Eurydice.*

### Circadian and Tidal Phenotypes Can Be Separated by Environmental Manipulation

**Figure 4:**
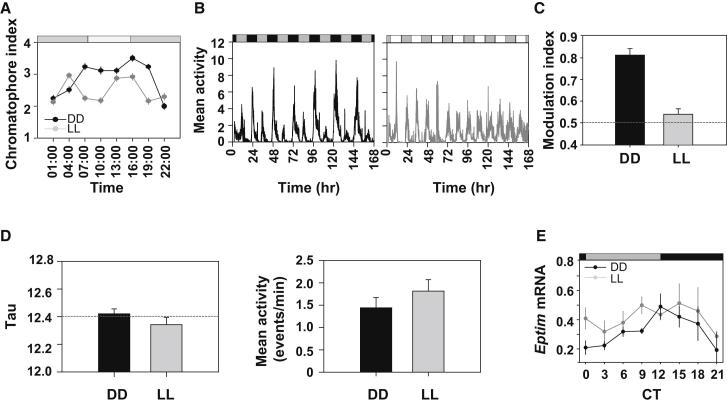
Circadian and Tidal Phenotypes Can Be Separated by Environmental Manipulations (A) Constant light (LL) disrupts the chromatophore rhythm (mean ± SEM) (white bar, subjective day; gray bar, subjective night). (B) LL (right) disrupts the amplitude modulation of tidal swimming (individual plots normalized to maximum activity) evident in DD (left). (C) Mean MI on DD and LL (mean + SEM; n = 21 and 26 for DD and LL, respectively; gray horizontal line, MI = 0.5). (D) Period of tidal swimming rhythm (left; gray line, 12.4 hr) and overall swimming activity (right) under DD and LL (mean + SEM; n = 30 for both LL and DD). (E) Expression of *Eptim* in heads of *Eurydice* held under DD or LL (mean ± SEM).

Bright constant light (LL) is an established means of generating an arrhythmic circadian profile. We therefore investigated the stability of tidal behavior of beach-caught *Eurydice* under LL or DD. The circadian chromatophore cycle was severely disrupted under LL (time × light interaction F_7,914_ = 34.4; Figure 4A) as was circadian modulation of tidal swimming (MI: DD = 0.81 + 0.03, LL = 0.54 + 0.03, F_1,45_ = 61.5, p ≈ 0; Figures 4B and 4C), consistent with their dependence on an underlying circadian oscillator. Importantly, the stability, phase, and period of tidal swimming were unaffected by LL (period F_1,58_ = 1.5, p = 0.23; Figure 4D). Also, the overall levels of swimming activity between the two conditions were not significantly different (F_1,58_ = 0.18, p = 0.67; Figures 4B and 4D). Thus, LL did not photoinhibit activity, but rather redistributed it equally between SD and SN tidal episodes. Consequently, the tidal and circadian phenotypes were dissociated, implying that tidal timekeeping does not require a competent circadian system.

To examine this dissociation at the molecular level, we tested the effects of LL on the *Eptim* mRNA cycle from tidally active, beach-caught animals (Figure 4E). Consistent with our earlier assays, DD expression was significantly rhythmic, whereas under LL *Eptim* levels did not vary with time (Figure 4E) but were elevated at time points corresponding to the trough of the DD cycle, consistent with derepression of a negative feedback loop (two-way ANOVA; time F_7,61_ = 2.25, p = 0.041; light F_1,61_ = 5.10, p = 0.027; interaction F_7,61_ = 0.44, p = 0.87; post hoc among DD time points, p < 0.05; LL, not significant). Consequently, three circadian phenotypes were disrupted in LL, physiological (chromatophore), behavioral (the MI), and molecular (*Eptim*), but tidal swimming remained unchanged under LL.

### Circadian and Tidal Phenotypes Can Be Separated by Molecular Manipulation

**Figure 5:**
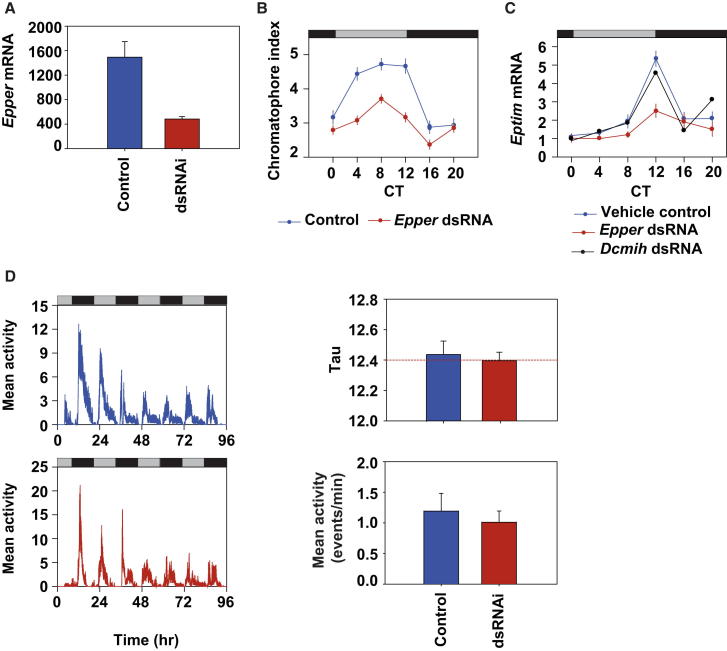
Circadian and Tidal Phenotypes Can Be Separated by Knockdown of *Epper* by RNAi (A) Knockdown of *Epper* by dsRNAi injections (mean ± SEM). (B) Mean chromatophore index (±SEM) for control (blue)- and *Epper* dsRNAi (red)-treated animals maintained in DD. Gray/black bars, subjective LD cycle. (C) Normalized *Eptim* transcript levels in DD (mean ± SEM) for vehicle control (blue), *Epper* dsRNAi (red), or control *Discoplax celeste* molt-inhibiting hormone (*Dcmih*) dsRNA controls (black). n = 3 for vehicle and *Epper* manipulation but n = 1 for *Dcmih*. There was no significant difference between *Epper* transcript levels in the heads of sham versus vehicle controls (t = 1.9, df = 9, p = 0.11). Gray/black bars, subjective LD cycle. (D) Tidal swimming period in DD is not altered by *Epper* dsRNAi. The left-hand panels show grouped swimming behavior of vehicle (blue)- and dsRNAi (red)-injected animals. Gray/black bars, subjective LD cycle. The right-hand panels show mean tidal period and mean activity levels (+SEM).

Finally, we attempted to separate tidal and circadian pacemaking using a molecular approach. Specifically, we targeted the circadian clock by RNAi knockdown of *Epper*, and we used the circadian cycle of *Eptim* expression as an independent molecular readout for the functional effect of knockdown on the circadian system. This was in preference to targeting *Eptim* because to do so would obscure our only molecular circadian phenotype. With direct thoracic injection of double-stranded RNAi (dsRNAi) *Epper*, we reliably reduced head *Epper* transcript to ∼20%–40% of normal levels 5–6 days postinjection (Figure 5A). Beach-collected animals were then screened for tidal swimming rhythms for 36 hr and were then injected, and their tidal and circadian rhythms were compared to sham-injected controls over the next few days. The chromatophore rhythm and *Eptim* mRNA cycle were both markedly damped by dsRNAi (Chromatophore rhythm: genotype F_1,749_ = 95.82 p ≈ 0; time F_5,749_ = 31.7, p ≈ 0; interaction F_5,749_ = 3.78, p = 0.002; Figure 5B; *Eptim* rhythm: time F_5,35_ = 18.5, p ≈ 0; genotype F_1,35_ = 15.85, p ≈ 0; interaction F_5,35_ = 4.47, p = 0.003; Figure 5C). Importantly, the reduction in the amplitude of the *Eptim* cycle means that the overall levels are considerably reduced, effectively providing an *Eptim* knockdown. Neither the sham- nor RNAi-injected animals, however, showed the normal levels of day-night modulation of swimming (MI sham = 0.68 + 0.04, RNAi = 0.64 + 0.04, F_1,27_ = 0.18, p = 0.67), so the injection itself compromises this phenotype (Figure 5D). Nevertheless, the circatidal swimming pattern was clear and sustained: both period and general swimming levels were completely unaffected by the RNAi (period F_1,36_ = 0.16, p = 0.69; activity F _1,36_ = 0.81, p = 0.38; Figure 5E). Therefore, the studies of LL and *Epper* RNA knockdown give similar conclusions: compromise of circadian timing by disrupting light input or by knockdown of a canonical clock factor had no effect on tidal periodicity. We therefore conclude that tidal timekeeping is independent of circadian timekeeping in *Eurydice* and that the tidal phenotype of *Eurydice* is driven by an autonomous circatidal pacemaker that can function independently of the EpPER and EpTIM circadian factors.

## Discussion

Using a combination of behavioral, physiological, and molecular approaches, we have dissected the complex temporal biology of *Eurydice pulchra.* Its adaptation to the intertidal environment is reflected in its circatidal rhythm of swimming, with the underlying tidal pacemaker possessing the three canonical properties of a biological clock: free-running period, entrainment to relevant environmental cues, and temperature compensation. Adaptation to the solar cycle is revealed by circadian rhythms of *Eptim* gene expression, chromatophore dispersion, and the day/night modulation of tidal activity. To test the long-standing hypothesis that the tidal clock is simply generated by an underlying circadian pacemaker, we exposed animals to LL or dsRNAi-mediated knockdown of *Epper* expression. Despite disruption of daily modulation of activity, and the circadian rhythms of chromatophore dispersal and *Eptim* expression, tidal behavior was completely refractory to these manipulations of circadian function. These results provide the first robust experimental demonstration that a circatidal clock is an independent timekeeping mechanism, distinct and separable from the circadian clock. It therefore resolves the long-standing dispute that was previously limited to inconclusive formal analysis of tidal and circadian rhythms [5, 6].

Our initial approach to dissociating tidal and circadian time was pharmacological, using inhibitors of CK1δ/ε. Based on studies in mammals [11], we expected that the period of circadian rhythms might be lengthened, but we had no prior assumption concerning any effect on tidal timing. Indeed, although circadian phenotypes (chromatophore and activity modulation) were altered as anticipated, there was also, surprisingly, a marked lengthening of period for tidal swimming activity. Setting aside the possibility of other, unknown drug targets, these observations could suggest that CK1ε plays a role in both tidal and circadian timekeeping. However, such prima facie evidence for a common mechanism of tidal and circadian clocks, perhaps through changes in EpPER stability [11], is counterbalanced by the LL and *Epper* RNAi studies, which revealed a clear dissociation between tidal and circadian machineries. Given the broad nature of CK1ε functions in mammals [19] CK1-mediated phosphorylation may contribute to both timing systems, but through very different substrates. Thus, it would appear that the circadian oscillator, as a module, does not determine tidal rhythmicity, but rather that CK1ε has a pleiotropic effect on tidal behavior.

Environmental and molecular dissociations of tidal and circadian timing were facilitated by our cloning of the circadian factors of *Eurydice*. This revealed a complement of canonical circadian genes comparable to those of fly and mouse, and functional tests in S2 cells confirmed the respective transactivational roles of EpCLK and EpBMAL1 at E-boxes and the negative regulatory properties of, in order of maximum potency, EpCRY2, EpPER, and EpTIM. This is reminiscent of the situation in monarch butterfly cell lines, in which CRY2 is the negative regulator role, with PER and TIM playing ancillary roles [20]. In contrast to the rhythmic expression of many, but not all, clock genes in *Drosophila* and mammals [21], *tim* was the only canonical clock gene of *Eurydice* with a circadian mRNA cycle, and its molecular rhythm was compromised by LL, a common feature of cycling clock gene mRNAs in LL in insects [22–24]. Even as the sole rhythmic negative element, *Eptim* could impose a circadian rhythm on the feedback loop. Moreover, cycling can be affected posttranscriptionally and posttranslationally [25], and proteomic surveys in mammals indeed reveal that the majority of cycling proteins do not have underlying rhythmic mature mRNAs [26, 27]. As *Epper* does not show mRNA cycles, the modest but characteristic EpPER cycle detected by the anti-EpPER serum observed in the two groups of neurons in each hemisphere would presumably be generated posttranscriptionally, perhaps as a reduction in EpPER stability during the light phase of the cycle, as occurs in *Drosophila* [28]. In common with other insects [29–31], and in contrast to *Drosophila*, these PER-ir cells were not PDH-positive cells [17]. In addition, as noted in several insects, including the silkmoth, *Antheraea pernyi*, which has eight PER-ir cells, EpPER-ir was mainly cytoplasmic with only weak nuclear signal [24, 31]. Nevertheless, work in S2 cells and the consequences of *Epper* RNAi for *Eptim* expression demonstrated that EpPER does have nuclear (transcriptional) functions, as also implied by transgenic rescue of behavioral rhythms in *per*^*01*^ flies, an experiment that spans the 420 million years of evolution between diptera and crustacea [32]. However, if EpCRY2 is the main negative regulator in *Eurydice*, as suggested by our S2 studies, new reagents will need to be developed to examine its spatiotemporal cellular distribution in the brain, with the expectation that nuclear-cytoplasmic movements would be a significant feature of EpCRY2 expression, as in lepidoptera [20].

The characterization of *Eptim* and *Epper* enabled us not only to establish a molecular assay of circadian timing, the rhythm of *Eptim* mRNA expression, but also develop RNAi of *Epper* as an experimental manipulation. Consistent with other studies of *per* knockdown in insects [33–35], this compromised circadian phenotypes of chromatophore dispersion and *Eptim* expression. Unfortunately, it was not possible to test its effect on activity modulation because the act of injection compromised the rhythm. A recent study of the mangrove cricket has also sought to examine whether knockdown of period by dsRNAi disrupts tidal and circadian rhythms of locomotion [35]. These insects show locomotor cycles, which have a tidal periodicity and an amplitude modulation of the tidal behavioral episodes, much like *Eurydice.* The authors have suggested that knockdown of period gene expression disrupts the modulation of locomotor rhythms but not the tidal periodicity, and they concluded that the circadian clock does not underlie the tidal clock [35]. However, scrutiny of the data presented suggests that *period* knockdown impacts negatively on both the putative circadian and tidal components. Furthermore, no compelling evidence is presented that the tidal rhythms are not submultiples of the circadian clock, for example, through the use of LL, which might be expected to disrupt the circadian component, but not the tidal one.

Consequently, a different interpretation of these data is that this insect evolved from a terrestrial ancestor that under light-dark entrainment had both morning (M) and evening (E) locomotor components of different amplitudes about 12 hr apart, as in *Drosophila* [36, 37]. This circadian rhythm would have adapted under tidal entrainment to the coastal region by slightly extending the M-E interval to 12.5 hr and thereby generating a circalunidian cycle of ∼25 hr. Thus, the underlying molecular tidal machinery would in effect be borrowed from the ancestral circadian clock. Under this scenario, *per* knockdown would be expected to have a general disruptive effect on both the ∼12.5 and 25.0 hr components, and indeed, this seems to be the case in the cricket. Consequently, we might imagine that different organisms could use different molecular solutions for generating tidal rhythmicity.

Therefore, across a range of phenotypes, it is clear that tidal timing can be expressed without an integrated circadian component and that suppression of circadian timing need not compromise tidal rhythms. Taken alongside our demonstration that tidal rhythms exhibit the defining properties of true biological clocks, we conclude that tidal timekeeping is independent of the expression of the circadian timekeepers *Epper* and *Eptim* in *Eurydice* and that the tidal phenotype of *Eurydice* is driven by an autonomous circatidal pacemaker. Finally, we note that in a simultaneous and very similar study to ours concerning the relationship of the circadian with the lunar clock of *Platynereis dumerilii*, manipulations of the circadian clock did not affect the lunar spawning cycle of this marine worm [38].

## Experimental Procedures

### Animal Collections and Behavioral and Chromatophore Recordings

*E. pulchra* were netted from Llanddona Beach, Anglesey, North Wales, UK at high water on spring tides, between April and November (2005−2012) and maintained in seawater in LD 12:12. Swimming was recorded in DAM10 *Drosophila* activity monitors (Trikinetics), and data were analyzed using ClockLab software (Actimetrics) [17]. Day/night modulation index of tidal swimming activity was analyzed using BeFLY [39]. Tidally rhythmic animals were snap frozen in liquid nitrogen at defined tidal and circadian times, and chromatophore patterns were imaged by digital camera and scored “blind” using the Hogben and Slome index [9]. Animals from nighttime high tides were placed immediately into swimming monitors and subjected to LL or DD at expected dawn. Recordings were initiated 24 hr after the last LD transition. Chromatophores were harvested at 3 hr intervals after 2 days in each condition. Heads were cropped and snap frozen for quantitative RT-PCR (qRT-PCR).

### PF670462 and PF4800567 Inhibitor Studies

The CK1ε/δ inhibitor, PF670462 (Tocris Biosciences) was dissolved in water. Freshly collected animals were individually placed in activity recording tubes in DD containing 2 ml of seawater and PF670462 at a final concentration of 25 μM. This was replaced with a second 25 μM dose 24 hr later at time of expected high water, thus minimizing disturbance. Animals were monitored for a further 5 days in DD before sampling at 3 hr intervals for chromatophore dispersion. PF670462 was also tested at final doses of 1 μM, 2.5 μM, 5 μM, and 50 μM for swimming behavior. The more specific CK1ε inhibitor PF4800567 (Tocris) was dissolved in DMSO to 50 μM and then diluted in seawater to 25 μM and 12.5 μM. Doses were administered as detailed for PF670.

### Cloning of cDNAs Encoding Canonical Clock Genes

Total RNA was extracted from heads, poly(A) mRNA was purified, cDNA was synthesized, and nested gradient PCRs were performed with degenerate PCR primers based on conserved regions of vertebrate and insect clock genes. Relevant amplicons were sequenced and 5′ and 3′ rapid amplification of cDNA ends (RACE) PCR amplifications performed to isolate the remaining 5′ and 3′ regions. An *E. pulchra* head cDNA library from circadian and tidal samples was also constructed and used to isolate the full-length *Eurydice period* (*Epper*).

### Quantitative RT-PCR

The expression of circadian clock genes was measured using Taqman MGB probes in qRT-PCR as described previously [17]. Data are expressed as either copy number for each transcript or as relative quantification, normalized to the reference gene *Eprpl32* (NCBI accession number CO157254.1).

### Phylogenetic and Sequence Analyses

Protein sequence homologs were retrieved from NCBI databases and FleaBase (http://wfleabase.org) for *Daphnia pulex*. Protein sequences were aligned with ClustalX2, and phylogenetic trees were constructed with the neighbor-joining method in MAGE 5 [40]. EMBL SMART (http://smart.embl.de) and NCBI CDD (http://www.ncbi.nlm.nih.gov/Structure/cdd/cdd.shtml) servers were used to detect and demarcate domains and motifs of clock proteins. The identity and similarity between proteins and domains/motifs (Table S1) were detected with the EMBOSS Pairwise Alignment Algorithms (EMBL-EBI).

### Tissue Distribution of Circadian Clock Gene Expression

The distribution of circadian clock gene transcripts and the reference gene, *Eprpl32* in brain, ventral nerve cord, hepatopancreas, gut, and ovaries was examined by standard RT-PCR.

### Antisera and Immunolocalization of Putative Oscillator Cells

Rabbit antisera for EpPER were raised against two synthetic peptides, conjugated to bovine thyroglobulin, which were affinity purified. Standard immunohistochemical procedures were performed on frontal head sections, followed by confocal microscopy and image analysis.

### S2 Cell Transcription Assays

*EpClk*, *Epbmal1*, *Epper*, *Eptim*, and *Epcry2* were amplified from their corresponding plasmids and subcloned into the *Drosophila* S2 cell expression vector pAc5.1/V5-HisA (Invitrogen). Similarly, *Epbmal1*Δ with a 36 residue C-terminal deletion was generated by PCR and subcloned into the vector. *Drosophila* S2 cells (Invitrogen) were maintained at 25°C, and luciferase activity was measured using the Dual Luciferase Reporter Assay Kit (Promega). Control transfections, including only reporter construct and empty vector (pAc5.1/V5-hisA), established baseline activity. Luciferase activity from transfected cells was normalized with *Renilla* activity. At least three independent transformations were performed for each assay.

### *Drosophila* Transformations

A fragment corresponding to the full-length *Epper* cDNA coding sequence was cloned into pUAST and injected into *w*^*1118*^ embryos (BestGene) to obtain multiple independently transformed lines. Male flies (∼3 days old) were entrained to LD 12:12 at 25°C for 4 days then allowed to free run in DD for 6 further days in Trikinetics monitors.

### Epper dsRNAi

A 758 bp dsRNA for *Epper* was synthesized using a DNA template corresponding to *Epper* sequence nt170–nt928. For the dsRNAi control, the molt-inhibiting hormone gene from the Christmas Island blue crab, *Discoplax celeste* (*Disco-mih*, NCBI accession number JF894386.1) was used. Double-stranded RNAs (200 ng) or elution buffer vehicle was injected into the hemocoel using pressure injection via glass microcapillaries. Gene suppression was assessed by qRT-PCR.

## Accession Numbers

The NCBI accession numbers of *Eurydice* genes are as follows: *Epper*, KC885967; *Epbmal1*, KC885968; *EpClock5*, KC885973; *EpClock1-4*, KC885976; *EpClock1-7*, KC885974; *EpClock1-9*, KC885975; *Eptimeless*, KC885969; *EpCK1e*, KC885972; *Epcry2*, KC885970; and *Ep6-4 photolyase*, KC885971.

## Supplemental Information

Document S1. Supplemental Experimental Procedures, Figures S1–S3, and Tables S1 and S2
