# Supplementary material for: Dissociation of Circadian and Circatidal Timekeeping in the Marine Crustacean Eurydice pulchra
Source: Curr Biol. 2013 Oct 7;23(19):1863–73. doi: 10.1016/j.cub.2013.08.038 (PMC3793863; doi:10.1016/j.cub.2013.08.038)
Supplement: Document S1. Supplemental Experimental Procedures, Figures S1–S3, and Tables S1 and S2 [file mmc1.pdf]

Current Biology, Volume *23*

Supplemental Information

Dissociation of Circadian and Circatidal Timekeeping in the Marine Crustacean *Eurydice pulchra*

Lin Zhang, Michael H. Hastings, Edward W. Green, Eran Tauber, Martin Sladek, Simon G. Webster, Charalambos P. Kyriacou, and David C. Wilcockson

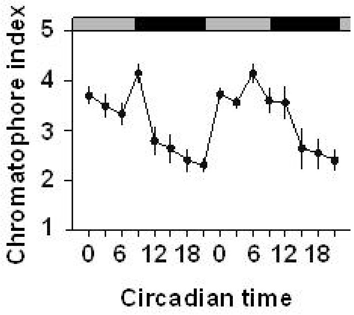

Figure S1. Free running rhythm of chromatophore dispersion, Related to Figure 1

Animals were maintained in constant darkness for two weeks, exposed to 5 days of LD12:12, then placed in DD and chromatophores scored in days 2 and 3 (means +/- sem).

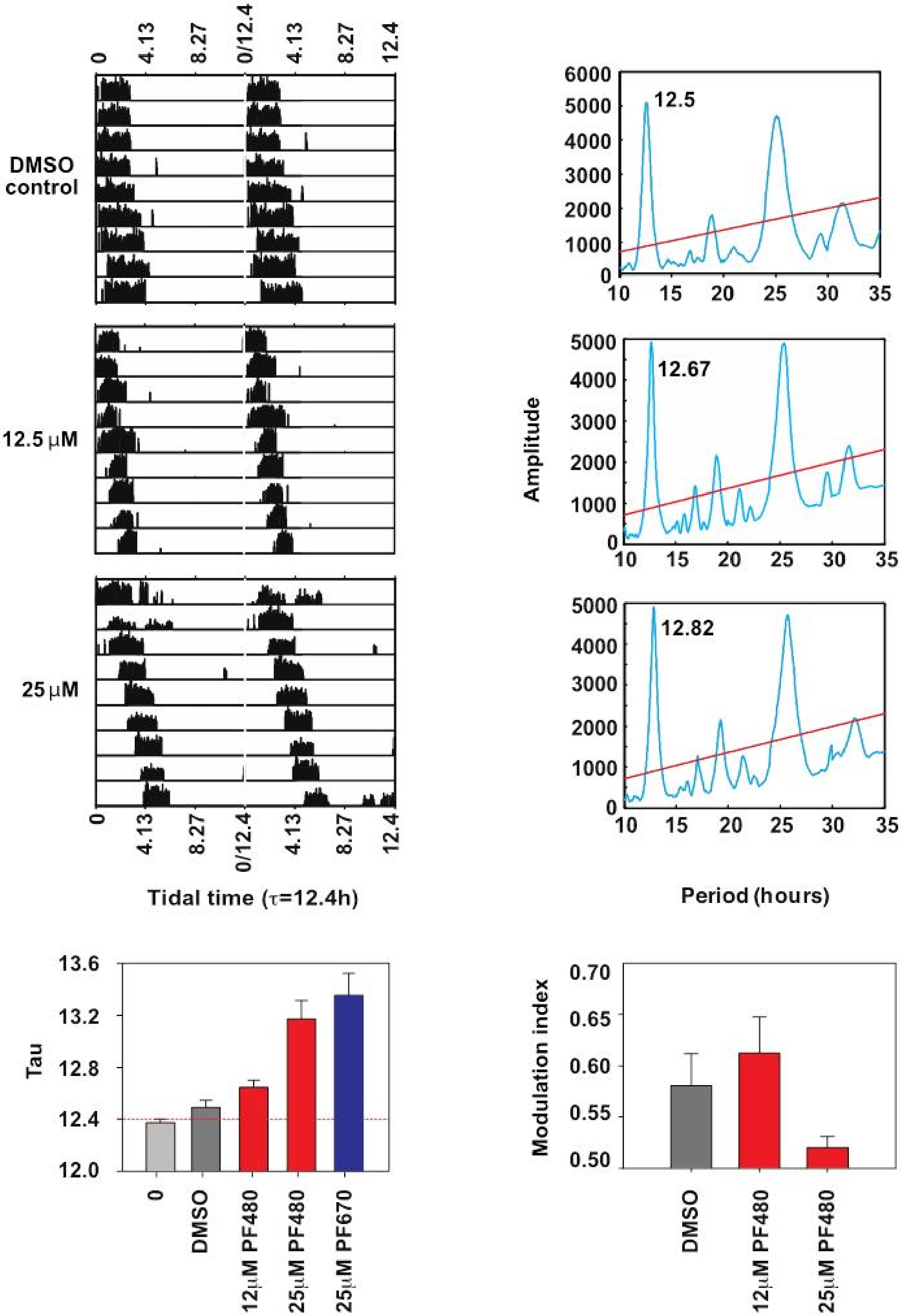

Figure S2. Casein kinase 1ε inhibition by PF4800567 lengthens tidal period, Related to Figure 2

Actograms are shown for control (DMSO) and 12.5 and 25 μM animals together with their associated periodograms (right-hand panels). The mean tidal periods (+sem) of DMSO and PF4800567 treated animals

compared to seawater controls and the animals treated with 25μM of PF670462 are shown in the bottom left-hand panel. ANOVA of tidal periods for the DMSO controls and the two doses of PF4800567 revealed a highly significant period lengthening ($F_{2,69}$=16.9 p=10^{-6}) compared to controls. DMSO controls however have a low modulation index (bottom right-hand panel) and while treatment with the highest dose of the inhibitor reduces this still further, the effect is not significant ($F_{2,58}$=2.35 p=0.105)

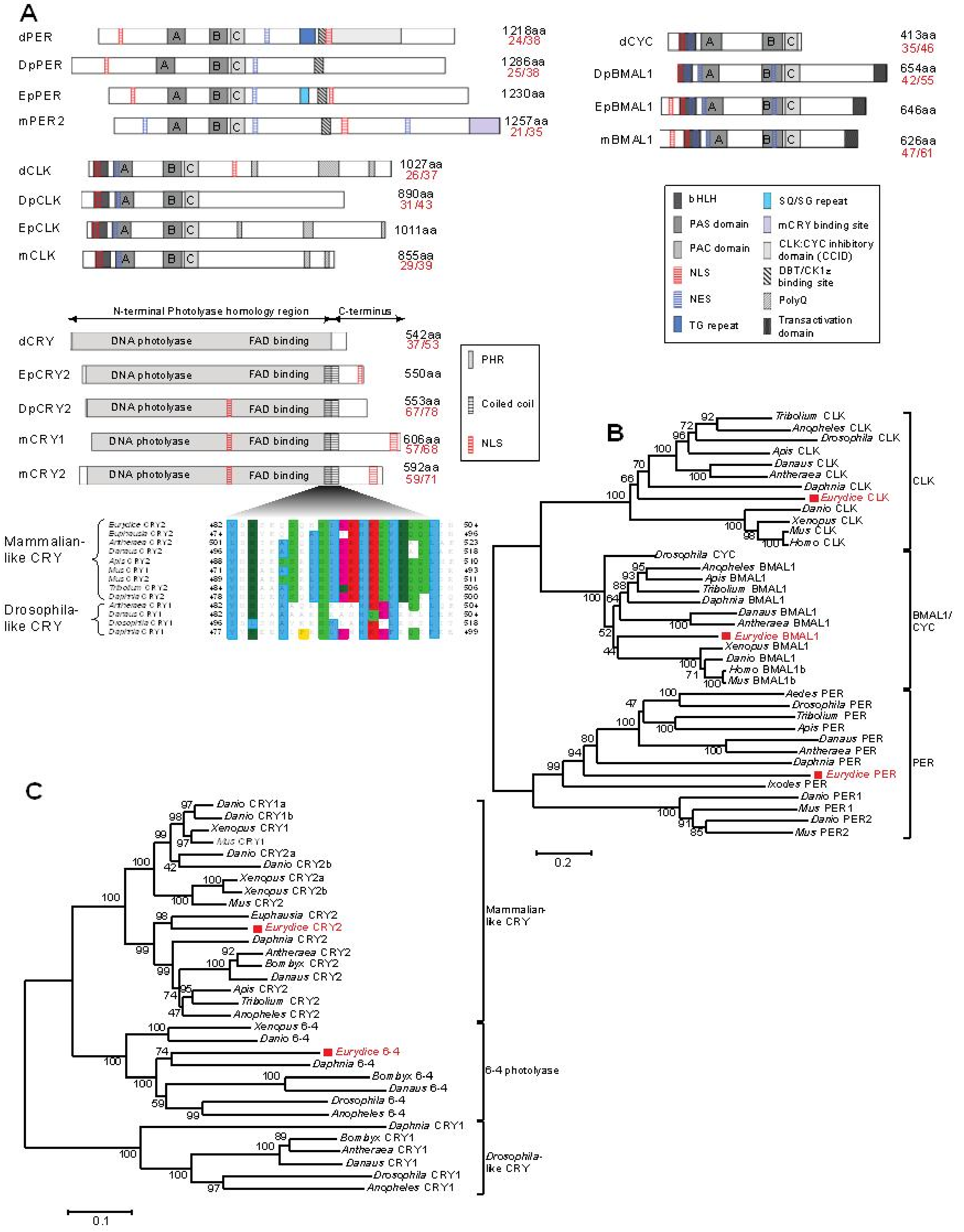
A
dPER 1218aa 24/38
DpPER 1286aa 25/38
EpPER 1230aa
mPER2 1257aa 21/35
dCLK 1027aa 26/37
DpCLK 890aa 31/43
EpCLK 1011aa
mCLK 855aa 29/39
dCYC 413aa 35/46
DpBMAL1 654aa 42/55
EpBMAL1 646aa
mBMAL1 626aa 47/61
bHLH
PAS domain
PAC domain
NLS
NES
TG repeat
SQ/SG repeat
mCRY binding site
CLK:CYC inhibitory domain (CCID)
DBT/CK1ε binding site
PolyQ
Transactivation domain
N-terminal Photolyase homology region
C-terminus
dCRY DNA photolyase FAD binding 542aa 37/53
EpCRY2 DNA photolyase FAD binding 550aa
DpCRY2 DNA photolyase FAD binding 553aa 67/78
mCRY1 DNA photolyase FAD binding 606aa 57/68
mCRY2 DNA photolyase FAD binding 592aa 59/71
PHR
Coiled coil
NLS
Mammalian-like CRY
Drosophila-like CRY
B
Tribolium CLK
Anopheles CLK
Drosophila CLK
Apis CLK
Danaus CLK
Antheraea CLK
Daphnia CLK
Eurydice CLK
Danio CLK
Xenopus CLK
Mus CLK
Homo CLK
CLK
Drosophila CYC
Anopheles BMAL1
Apis BMAL1
Tribolium BMAL1
Daphnia BMAL1
Danaus BMAL1
Antheraea BMAL1
Eurydice BMAL1
Xenopus BMAL1
Danio BMAL1
Homo BMAL1b
Mus BMAL1b
BMAL1/CYC
Aedes PER
Drosophila PER
Tribolium PER
Apis PER
Danaus PER
Antheraea PER
Daphnia PER
Eurydice PER
Ixodes PER
Danio PER1
Mus PER1
Danio PER2
Mus PER2
PER
0.2
C
Danio CRY1a
Danio CRY1b
Xenopus CRY1
Mus CRY1
Danio CRY2a
Danio CRY2b
Xenopus CRY2a
Xenopus CRY2b
Mus CRY2
Euphausia CRY2
Eurydice CRY2
Daphnia CRY2
Antheraea CRY2
Bombyx CRY2
Danaus CRY2
Apis CRY2
Tribolium CRY2
Anopheles CRY2
Mammalian-like CRY
Xenopus 6-4
Danio 6-4
Eurydice 6-4
Daphnia 6-4
Bombyx 6-4
Danaus 6-4
Drosophila 6-4
Anopheles 6-4
6-4 photolyase
Daphnia CRY1
Bombyx CRY1
Antheraea CRY1
Danaus CRY1
Drosophila CRY1
Anopheles CRY1
Drosophila-like CRY
0.1

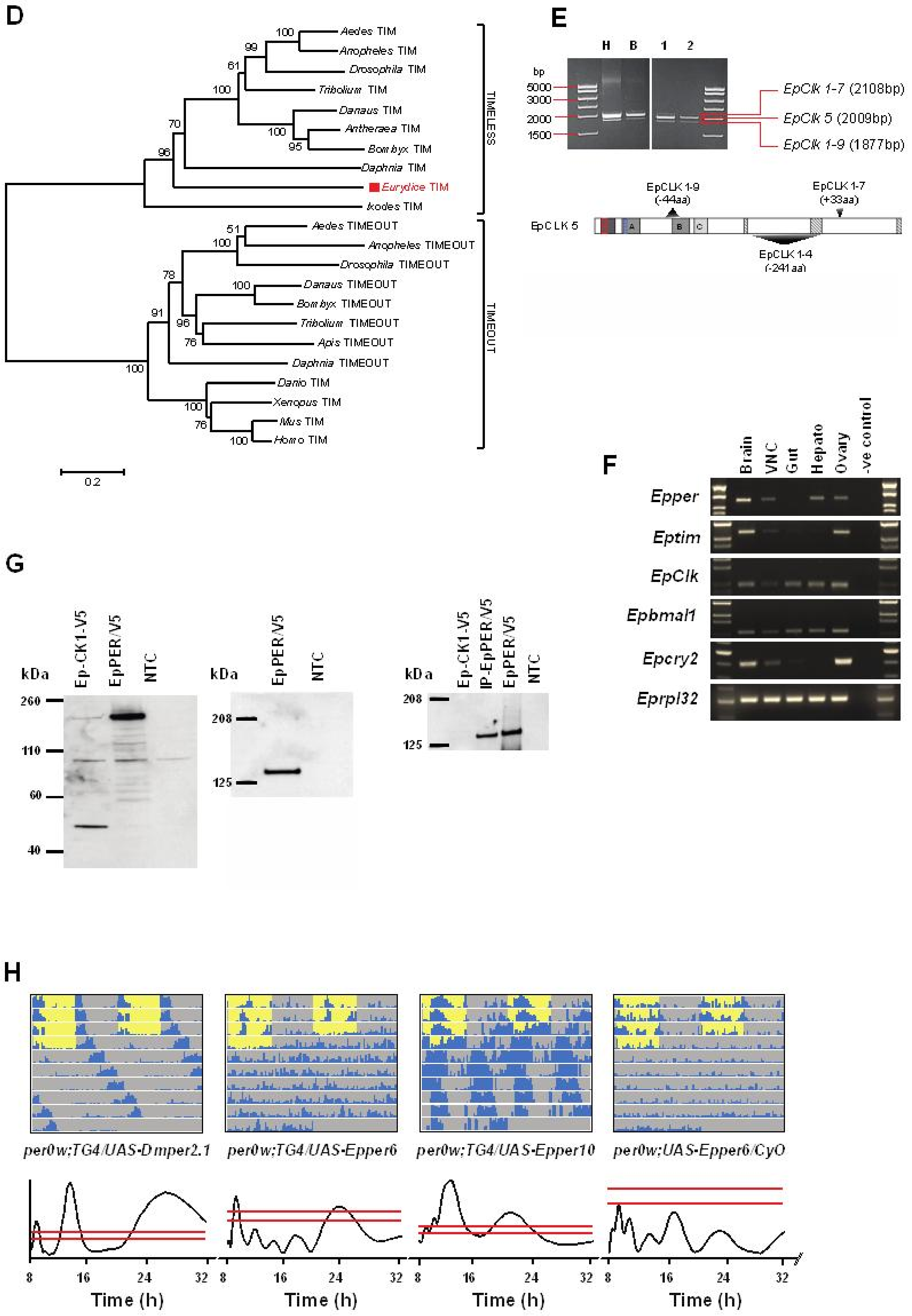
D
Aedes TIM
Anopheles TIM
Drosophila TIM
Tribolium TIM
Danaus TIM
Antheraea TIM
Bombyx TIM
Daphnia TIM
Eurydice TIM
Ixodes TIM
Aedes TIMEOUT
Anopheles TIMEOUT
Drosophila TIMEOUT
Danaus TIMEOUT
Bombyx TIMEOUT
Tribolium TIMEOUT
Apis TIMEOUT
Daphnia TIMEOUT
Danio TIM
Xenopus TIM
Mus TIM
Homo TIM
TIMELESS
TIMEOUT
0.2
E
H B 1 2
bp
5000
3000
2000
1500
EpClk 1-7 (2108bp)
EpClk 5 (2009bp)
EpClk 1-9 (1877bp)
EpCLK 1-9 (-44aa)
EpCLK 1-7 (+33aa)
EpCLK 5
EpCLK 1-4 (-241aa)
F
Brain
VNC
Gut
Hepato
Ovary
-ve control
Epper
Eptim
EpClk
Epbmal1
Epcry2
Eprpl32
G
kDa
Ep-CK1-V5
EpPER/V5
NTC
IP-EpPER/V5
260
110
60
40
208
125
H
per0w;TG4/UAS-Dmper2.1
per0w;TG4/UAS-Epper6
per0w;TG4/UAS-Epper10
per0w;UAS-Epper6/CyO
Time (h)

Figure S3. Molecular analysis of putative clock genes in *Eurydice pulchra*, Related to Figure 3

A. *Eurydice* clock protein domains compared to orthologues from *Daphnia pulex* (Dp), mouse and *Drosophila*. Protein length and % identity/similarity in red. C-terminal CRY sequences that distinguish *Drosophila*-like CRY and mammalian-like CRY are expanded. The CRY C-terminal coiled-coil region important for CRY-mediated repression is expanded.

B. Neighbour-joining tree reflecting the phylogeny of PAS domain clock proteins PER, CLOCK and BMAL1/CYC. *Eurydice* sequences in red.

C. *Eurydice* CRY2 clusters with the mammalian CRY like molecules not the Drosophila-like CRYs**.**

D. EpTIM is a TIMELESS not a TIMEOUT/TIM2 orthologue**.**

E. RT-PCR of *EpClk* showing the most abundant (EpCLK5) isoform *EpClk5* expression in heads (H) and bodies (B) and whole animal (lanes labelled 1 and 2). DNA size markers are also given on each gel. The cartoon below gives a schematic representation of *EpClk* splice isoforms.

F. Circadian clock gene transcripts are expressed widely in *Eurydice*. *Eprpl32* was consistently expressed in all tissues. Negative control reactions lacked RT enzyme in the cDNA synthesis. VNC, ventral nerve cord; Hepato, hepatopancreas.

G. Left-hand panel, Western blot of COS-7 cell extracts probed with anti-V5 serum reveal expressed V5-tagged EpPER and EpCK1. Central panel, Western blot of COS-7 cell extracts probed with anti-EpPER serum (1 in 5,000) reveals expressed V5-tagged EpPER in transfected but not in non-transfected cells. Right-hand panel. Western blot of COS-7 cell extracts probed with anti-EpPER serum (1 in 5,000) directly or after immunoprecipitation of extract with anti-V5 serum (IP) to isolate EpPER-V5. Note absence of EpPER signal in non-transfected cells (NTC) and cells transfected with EpCK1-V5.

H. *Eurydice per* functions as a clock component in transgenic *D. melanogaster***.** Representative actograms of *Drosophila* per^{01} mutants transformed with *Epper* that show circadian locomotor patterns. Left-hand panel, double-plotted histogram of positive control per^{01}*w; TG4 (timgal4)/UAS-Dmper2.1* transformant in 4 days of LD12:12 then 6 days of DD. Panel below is a spectral analysis of the DD data. Y-axis, spectral density, X-axis, period. Lower/upper horizontal red lines show

95/99% confidence limits. Adjacent panels show individuals from two independent transformant lines expressing *UAS-Epper*. Right panel, $per^{01}w$ arrhythmic negative control. Quantitative analysis of rhythmicity appears in Table S2.

Table S1. Comparison of amino acid identity/similarity (%) among PER, CLK, BMAL1/CYC, TIM and CRY, Related to Figure 3

	EpPER vs DpPER	EpPER vs dPER	EpPER vs mPER2	
**Whole protein**	25/38	24/38	21/35	
**PAS-A**	37/59	43/57	24/41	
**PAS-B**	52/65	44/58	29/46	
**PAC**	58/74	48/71	46/66	
**C-terminus**	22/34	20/31	20/32	
	**EpCLK vs DpCLK**	**EpCLK vs dCLK**	**EpCLK vs mCLK**	
**Whole protein**	31/43	26/37	29/39	
**bHLH**	61/77	65/82	61/75	
**PAS-A**	57/72	44/57	48/64	
**PAS-B**	77/86	67/83	73/83	
**PAC**	77/89	71/93	86/98	
**C-terminus**	20/31	14/19	14/21	
	**EpBMAL1 vs DpBMAL1**	**EpBMAL1 vs dCYC**	**EpBMAL1 vs mBMAL1**	
**Whole protein**	42/55	35/46	47/61	
**bHLH**	52/67	72/83	70/83	
**PAS-A**	79/91	71/85	71/88	
**PAS-B**	58/66	50/77	51/68	
**PAC**	48/73	50/68	52/75	
**BCTD**	81/86		81/86	
**C-terminus (without BCTD)**	19/35		23/38	
	**EpTIM vs DpTIM**	**EpTIM vs dTIM**		
**Whole protein**	25/38	23/37		
**TIS-1**	32/44	47/63		
**TIS-2**	35/57	47/64		
**CLD**	13/23	16/28		
	**EpCRY2 vs DpCRY2**	**EpCRY2 vs dCRY**	**EpCRY2 /mCRY1**	**EpCRY2 /mCRY2**
**Whole protein**	67/78	37/53	57/68	59/71
**PHR**	73/84	38/55	70/82	67/80
**Coiled coil**	70/83		61/87	61/87
**C-terminus**	24/32	16/31	10/13	17/24

BCTD, BMAL1 C-terminal Transactivation Domain (1); C-terminus was defined as the entire peptide sequence downstream of PAC domain in PER, CLK and BMAL1 or downstream of PHR domain in CRY2; TIS-1/TIS-2, TIM Interaction Sites with PER and CLD, Cytoplasmic Location Domain in TIM (2) PHR, Photolyase Homology Region; Coiled coil region was defined as in (1).

Table S2. Locomotor activity rhythms of transgenic flies in constant darkness, Related to Figure 3

Transgenic line	N	N(sr)	N(mr)	N(ar)	Rhy (%)	Period(h)	SD	SEM
wper⁺;UAS-Dmper2.1/CyO	32	31	0	1	96.9	24.65	1.99	0.36
wper^{01};TG4/UAS-Dmper2.1	32	32	0	0	100	26.60	1.44	0.26
wper^{01};TG4/UAS-Epper6	**58**	**18**	**0**	**40**	**31.0**	**26.93**	**2.76**	**0.65**
wper^{01};TG4/UAS-Epper10	**30**	**11**	**3**	**16**	**36.7**	**23.56**	**4.30**	**1.30**
wper^{01};TG4/CyO	30	8	5	16	26.7	16.87	6.25	2.21
wper^{01};UAS-Epper6/CyO	31	1	0	30	3.3	24.48		
wper^{01};TG4/CyO	59	4	5	50	6.8	22.58	10.09	5.04
wper^{01};UAS-Epper10/CyO	32	4	5	21	12.5	18.38	5.71	2.86

Results of locomotor rhythm analyses in *D. melanogaster* transformants carrying a *UAS-Epper* transgene (two independent lines, 6 and 10, both inserted on chromosome 2). *CyO* is a second chromosome balancer, *TG4* is a *tim-gal4* driver on chromosome 2. Significant rhythms by both spectral analysis (beyond 1%) and autocorrelation (5%) are indicated for flies that gave a dominant single rhythm, N(sr), multiple rhythms, N(mr), or were arrhythmic, N(ar).

SUPPLEMENTAL EXPERIMENTAL PROCEDURES

Animal collections and behavioural recordings

Eurydice pulchra were maintained in fresh seawater at ambient seawater temperature in an aquarium in 20L plastic containers on a 12:12LD cycle. For sampling of tidally rhythmic animals for qRT-PCR, beach-caught animals were removed to the laboratory and placed immediately into activity monitors. Swimming was recorded in modified DAM10 *Drosophila* activity monitors using 11 mm diameter tubes (Trikinetics, Waltham MA) and in constant darkness (DD) as described previously (3). Swimming activity was recorded 10 times per second and counts pooled into 1 minute 'bins'. Tau was determined by Chi Squared periodogram analysis with significance level set at P<0.001. Day/night modulation of tidal swimming activity was analysed using the BeFLY! Suite of programmes developed in Leicester (4). Total amount of activity in each night time tidal episode was divided by the amount of activity of the successive daytime episode plus the preceding night episode to generate a modulation index (MI) that was usually between 0.7 and 0.8 from beach-caught animals. For the temperature compensation experiments beach-caught animals were removed to activity monitors held in DD at 11°C, 17°C (ambient seawater temperature) and 21°C and swimming behaviour recorded for 6 days.

Chromatophore dispersion rhythms

Rhythms of chromatophore dispersion were analysed by snap freezing animals in liquid nitrogen to preserve chromatophore morphology, followed by digital imaging under a dissecting microscope. Chromatophores from the dorsal thorax, abdomen and lateral abdomen were staged according the Hogben and Slome index (5) which was modified to include 0.5 point scoring intervals. All scoring was performed blind by an independent experimenter.

Effect of light regimes regime on swimming, chromatophore and gene expression rhythms

Animals were removed from the shore at night-time high tides and placed immediately into activity monitors. At time of expected dawn monitors were subjected to LL or maintained in DD for 24 h before swimming was recorded. To assess the effect of LL and DD on chromatophore rhythms, animals were

entrained under LD12:12 for 5 days but kept in 400mL conical flasks containing fresh seawater and with sand to a depth of 2mm in groups of 20-30 animals. Chromatophores were harvested at 3h intervals on the second and third day in each condition. For circadian gene expression studies, heads were cropped and snap frozen for qRT-PCR after previous entrainment in LD cycles and one full day in DD.

Cloning of cDNAs encoding canonical clock genes

Total RNA was extracted from animal heads with TRIzol (Invitrogen), and Poly(A) mRNA was purified using Dynabeads oligo $(dT)_{25}$ (Dynal). After first-strand cDNA synthesis**,** a two-round nested gradient PCR reaction was performed with degenerate PCR primers (for sequence details see below) based on conserved regions of vertebrate and insect clock genes. Amplicons of the predicted sizes were cut and purified from the gel, sub-cloned, and sequenced. The remaining 5' and 3' regions of the cDNA fragments were isolated with 5'/3' Race System for Rapid Amplification of cDNA Ends (Invitrogen) and 5'/3' Race kit (Roche Applied Science). An *E. pulchra* head cDNA library from circadian and tidal samples was also constructed from poly(A) mRNA using Stratagene's cDNA synthesis kit and lambda Uni-Zap XR vector and used to isolate the full-length *Eurydice period (Epper).*

Semi-quantitative RT-PCR of EpClks

Total RNA was extracted with same method as above from pooled heads or bodies and individual animals and then treated with Ambion DNase-free kit to remove DNA contaminations. 50-100ng DNase treated total RNA was used to synthesise first-strand cDNA with SuperScript II Reverse Transcriptase (Invitrogen). Semi-quantitative PCR was carried out for 25 cycles with a pair of *EpClk* co-specific primers (see below) and Expand High Fidelity DNA polymerase (Roche Applied Science).

Quantitative reverse transcription PCR

The expression of circadian clock genes and candidate tidal genes was measured using Taqman™ MGB probes in quantitative reverse transcription PCR (qRT-PCR) as described previously (3) . Hydrolysis probes were designed using Applied Biosystems (Life Technologies, NY) Primer Express 3 software and purchased from Life Technologies. Probes were labelled with FAM fluors and used with Sensimix Probe II qPCR reagents (Bioline,

London, UK). Reverse transcription was done on total RNA extracted with Trizol reagent (Invitrogen, Life technologies, NY) and DNAse treated using *DNAse Free* reagents (Ambion, UK). RT reagents (Taqman™ High-Capacity cDNA Reverse Transcription) were purchased from Applied Biosystems and 500ng – 1µg RNA was reverse transcribed using random hexamer primers for 2h at 37°C. Standard curves for qPCR were produced *in vitro* from a PCR-generated DNA template containing a T7 phage promoter site (see primer sequences below) incorporated via the forward primer and using Megascript transcription reagents (Ambion, UK). Complementary RNA was polyacrylamide gel purified (6M urea-PAGE, 10%), products of the expected size excised and eluted in probe elution solution (Ambion) and quantified by spectrophotometry (Nanodrop, Thermo Fisher Scientific, Leicestershire, UK) and copy numbers determined empirically (as detailed in ref 3). cRNA was serially diluted in the range 10^9 copies to 10^3 copies per reaction and reverse transcribed. Data are expressed as either copy number for each transcript or as relative quantification normalised to the reference ribosomal protein gene *Eprpl32.*

Phylogenetic and sequence analyses

The protein sequences homologous to PER, TIM, CLK, CYC/BMAL1, CRY and 6-4 Photolyase of other organisms were retrieved from NCBI databases. For the water flea *Daphnia pulex*, protein sequences were taken from Genes 2010 Beta 3 database in FleaBase (http://wfleabase.org). Protein sequences were aligned with ClustalX v2 (6), and adjusted using Jalview v2.4 (7). Phylogenetic trees were constructed with Neighbour-Joining (NJ) method as implemented in MEGA 5 (8). Partial/pairwise deletion and Poisson corrected distances were performed, with 1000 bootstrap replications. The species names and accession numbers of sequence data used in this analysis are supplied below. EMBL SMART (http://smart.embl.de/) (9) and NCBI CDD (http://www.ncbl.nlm.nih.gov/Structure/cdd/cdd.shtml) (10) servers were used to detect and demarcate domains and motifs of clock proteins. We used NLStradamus (11) and NLS Mapper (12) for nuclear localisation signal prediction and NetNES (13) as well as consensus sequences for identifying nuclear export signals (NES). The identity and similarity between proteins (Table S1) and domains/motifs were calculated with EMBOSS Pairwise Alignment Algorithms (EMBL-EBI).

Constructs and S2 cell transcription assays.

The constructs for *Drosophila* clock genes, pAct-*dClk*, pAct-*dper*, pAct-*dtim* and pAC5.1-*dcry/*V5-His were kindly provided by Steven Reppert (University of Massachusetts, USA), as was the *Drosophila* E-box luciferase reporter construct, pGL3 *4E-hs-luc*. The latter has four tandem repeats of the *dper* E-box *(CACGTG)* with 18 bp of immediate flanking sequence, fused with a minimal *hsp70* promoter upstream of *luciferase* (14). pCopia-*Renilla* was a gift from Michael Rosbash (Brandeis University).

Drosophila S2 cells (Invitrogen) were maintained at 25°C in HyClone SFX-insect medium (Thermo Scientific). Transfection was carried out using Cellfectin reagent (Invitrogen). Cells were incubated at 25°C for 48 h after transfection and lysed with passive lysis buffer (Promega). Luciferase activities were measured using Dual Luciferase Reporter Assay Kit (Promega) and FLUOstar Omega (BMG Labtech) microplate reader. For each assay, a control transfection, including reporter construct E-box (pGL3 *4E-hs-luc*), pCopia-*Renilla* and empty vector (pAc5.1/V5-hisA) were used to establish the baseline activity. Luciferase activity from transfected cells was normalised with *Renilla* activity. At least three independent transformations were performed for each assay.

Drosophila transformations

UAS-Dmper2.1 (P{Per}2-1) and *tim-Gal4 (TG4)* (15, 16) were obtained from the Bloomington stock centre. A NotI - XbaI fragment corresponding to the full-length *Epper* cDNA coding sequence was cloned into the pUAST vector (17). The pUAS-*Epper* construct was injected into w^{1118} embryos through a transgenic service (BestGene Inc). Multiple, independently transformed lines with balancer were generated and mapped to individual chromosomes. For rescue of the per^{01} flies, per^{01}*; tim-Gal4 (TG4)* was crossed with independent transformed lines *UAS-Epper6* and *UAS-Epper10* (both transgene inserted on second chromosome), respectively. *TG4-UAS-Dmper2.1* and per^{01} were used as controls. Male flies (~3 days old) were entrained to light: dark cycles (LD12:12) at 25˚C for 4 days and then activity was monitored in the constant darkness for 6 further days using the Trikinetics DAM10 system (Waltham, Mass). Locomotor activity measurement and spectral analysis and autocorrelation have been described previously (18).

COS7 cells.

To study the specificity of the EpPER antisera, COS-7 cells transfected with either V5-tagged *Epper* alone, or empty vector were lysed 24 hours after transfection in lysis buffer (150mMNaCl/ 20mM HEPES [pH 7.5]/ 0.1% Nonidet P-40/ 1mM EDTA/ 2mM DTT) supplemented with Complete protease inhibitor mixture (Roche Applied Science). The cells were then mechanically sheared, and lysates were centrifuged at 16,000 g for 10 min at 4°C. Total protein (20 μg) was resolved in SDS/PAGE and transferred to nitrocellulose membrane (Bio-Rad). Membranes were blocked with 2% ECL blocker (Amersham Biosciences) in Tris-buffered saline and then incubated overnight in TBS containing either affinity purified anti-EpPER antisera diluted 1:5000 or anti-V5 antibody (Life technologies) 1:10000. Subsequently, membranes were washed, incubated in relevant secondary antibodies (Sigma) and subjected to chemiluminescence detection using ECL plus reagent (Amersham Biosciences). Both anti-V5 and anti-EpPER antibodies revealed a single specific band around 130kDa.

Epper dsRNAi

A 758 bp double-stranded RNA for *Epper* was synthesized using a DNA template corresponding to *Epperiod* sequence nt170-nt928. PCRs were primed using oligonucleotides containing a T7 phage promoter region (see below) so as to incorporate the promoter sequence in the 5' and 3' directions in separate reactions. Single stranded cRNA in both directions was synthesized using a Megascript RNAi kit (Ambion, UK) according to the manufacturer's instructions and eluted in the supplied injection buffer (10mM Tris-HCl pH 7, 1mM EDTA). Complementary RNA strands were subsequently hybridized by heating to 75°C (5 minutes) and cooling to room temperature. Contaminating single stranded RNA and DNA template were removed by *RNAse* and *DNAse 1* treatment and column purified, all according to the manufacturer's protocols. Double stranded products were then analysed for size and purity by agarose gel electrophoresis, quantified by spectrophotometry (Nanodrop ND-1000) and the concentration adjusted to 1μg per μL. For the dsRNAi control the moult-inhibiting hormone gene from the nervous tissue of the Christmas Island blue crab, *Discoplax celeste* (*Disco-mih*, accession number JF894386.1) was used to generate a 489bp dsRNA in the same was as described above. Double-stranded RNAs or elution buffer vehicle was injected into the haemocoel between anterior tergites through a finely pulled glass capillary and using compressed nitrogen delivered by a PV830 PicoPump (World Precision Instruments, FL)

set at 40psi. Injections delivered 200nL (200ng) dsRNA or vehicle over a 60s period. Animals were held out of water for 2-3 minutes to ensure injected fluids did not leak out of the puncture wound. Gene suppression was assessed by qRT-PCR as described above, except that the reverse transcription reaction was primed with oligodT$_{(15)}$ primer instead of random hexamers.

Tissue distribution of circadian clock gene expression

Animals maintained in the absence of tidal cues and held in DD were chilled in ice-cold seawater before dissection. Tissues including brain, ventral nerve cord (VNC), hepatopancreas, gut and ovaries were removed using sharpened watch-makers forceps in ice-cold nuclease-free physiological saline and snap frozen. Pooled samples of each tissue were subject to RNA extraction using Trizol and RT-PCR. Briefly, 1µg total RNA was reverse transcribed using Taqman High Capacity cDNA RT reagents (Applied Biosystems) in 20µL reaction volume and using random hexamer primers. 1µL of each cDNA was used in a 25µL PCR reaction containing 0.5µM each primer and amplified using Amplitaq Gold PCR mastermix (Applied Biosystems). Primer sequences for each gene are detailed below. Cycling conditions were: 94°C 9mins; 35 cycles of 94°C 1min, 55°C 30s, 72°C 45s, followed by a final extension at 72°C for 7 minutes. Amplicons were examined on a 2% agarose gel. To confirm that each amplicon was specific for the gene of interest, products were ligated into a PCR 4- TOPO vector and transformed (Top-10F', Invitrogen). Plasmid DNA from positive clones containing inserts of correct sizes were purified and sequenced.

Antisera and immunolocalisation of putative oscillator cells

Antisera for EpPER were raised against 2 synthetic peptides (15 amino acids) predicted to be antigenic and represent hydrophilic hairpin loops on the EpPER protein (designed by SigmaGenosys, UK) with the following sequences: RSNPKTVSSDESGDSK[C] found near the middle of the protein sequence with four predicted beta turns and [C]SGPDDKSDEEDSPS located towards the C-terminus of the PER protein and predicted to contain five beta turns. Each peptide was conjugated to bovine thyroglobulin using 3-maleimidobenzoic acid *N*-hydroxysuccinimide ester, facilitated by the addition of the cystine residue added to each peptide as shown above in parentheses. Conjugates were co-injected into 2 New Zealand White rabbits which produced sera SG1993 and SG1994. Five immunizations were done at 14 day intervals after which terminal exsanguinations

were performed. The first immunisation was done with 200μg peptide in complete Freund's adjuvant and thereafter with 100μg in incomplete Freund's. All peptide synthesis, conjugation and animal procedures were performed as a commercial service by Sigma Genosys, Haverhill, UK.

Serum SG1994 was affinity purified using both antigens coupled to CNBr-Activated Sepharose 4B (Amersham Pharmacia, UK). 1g CNBr activated Sepharose resin was pre-treated and swollen with 1mM HCl for 1 hour at RT and 5mg of each peptide coupled overnight in 5mL coupling buffer (0.1M $NaHCO_3$, 0.5M NaCl, pH8.0) at 4°C. After washing with coupling buffer and blocking with 200mM glycine (pH8.0) for 1h at RT and further washes in PBS + 0.05% Tween20, 5mL raw serum, was passed repeatedly over the resin in a 20mL syringe cartridge for 2h at RT. Following 3 washes with PBST the bound antibody was eluted with 100mM glycine (pH2.5) and immediately neutralised with 0.1M Na-acetate, 0.5M NaCl (pH4.0) at a ratio of 50:1 eluate. Eluates were concentrated with Microcon YM30 spin columns (Millipore, Billerica, MA) to a final concentration of ~2mg/mL. Animals for ICC were harvested at the appropriate time according to the experimental regime. Heads were taken following rapid decapitation and placed immediately in Bouin's-Hollande fixative for 12h at 4°C and washed repeatedly in 50% ethanol until leaching of picric acid had ceased. Tissues were dehydrated through ethanol series, cleared in methyl benzoate until sunk and washed in toluene before embedding in paraffin wax. Serial frontal sections at 7μm thickness were cut and mounted on glycerine albumin treated slides. Sections were gradually rehydrated through an ethanol series to PBS before blocking in 10% normal sheep serum for 1h and primary antisera diluted in PBST (PBS+0.05% Tween 20) applied and incubated overnight at 4°C. Following incubation and 3x10 minute washes in PBST, secondary antisera were applied for 1h at RT followed by washes in PBST (3x10min) and mounting under a coverslip with Vectorshield-H1000 mounting medium (Vector Laboratories, Burlingame, CA). For DAPI counterstaining, DAPI was diluted to 300nM in PBS and slides incubated for 2 min at RT before washing in PBS and mounting. Antisera were used at the following dilutions: SG1994 (affinity purified) 1:1000 (2μg/mL), Secondary antisera, Goat anti rabbit Alexa 568 (Molecular probes, Life Technologies Paisley, UK) was used at 1:500

Specificity controls for the EpPER sera were performed by preabsorbing 20μL anti-EpPER sera with 5μg of each peptide antigen diluted in PBS and incubated overnight at 4°C . Sections were prepared and antisera

applied as described above but visualised using the peroxidase-antiperoxidase method (19). Sections were dehydrated and mounted under DPX for light microscopy. Preabsorbtion with antigen abolished all staining (Figure S3~~S1~~).

Images were obtained using either a Zeiss LSM510 or Leica TCS SP5 laser confocal microscope and processed using ImageJ and Adobe Photoshop CS4 software. Immunostaining intensity was analysed digitally using ImageJ. Positively stained cells were selected manually and intensity readings recorded. Background staining was assessed at three separate points on each image and the mean of these subtracted manually from the region of interest.

Statistical analysis

Statistical analyses of data was performed using STATISTICA (data analysis software system), version 8.0. StatSoft, Inc. (2007).

Primers and probes

	Primer name	Forward sequence (5’→3’)	Reverse sequence (5’→3’)	Taqman probes
Taqman qPCR	Epper_Tq	GCCCGTGACATCGTACCAA	CCAAAGTTCGACGGCGTTT	6-FAM-CCTTGTAAGGAACCGATGG
	Eptim_Tq	TGGTCAGCCACATCAAACAGTT	GACGCGCCCATATTGCA	6-FAM-CCACCATGGATATCAT
	EpClk_Tq	CGGAACTGCAAGACCAATCC	CTCGTTTCCATGGCGTTGT	6-FAM-CCTCTCCAGCTTCC
	Epbmal1_Tq	GATTGTGCGTCTTCCCAGTCA	CCTCCTGGCGAGTTGATGA	6-FAM-TCGGCAACATCAGTGAA
	Epcry2_Tq	ACATGCTGTCGCTTGTTTTCTC	TGCCCTCTTCCCAGGATACC	6-FAM- TCGCGGAGACCTTT
	EpCk1e_Tq	GAGGCGAGTCATTACGGATCA	CCACTGCTTCTCTGACTCTATTCG	6-FAM- CAGTAACGAGCGGCTG
	Eprpl32_Tq	CTGCGGAGAAATCGCACAT	TTTGCTCTTTCCACGATCGA	6-FAM-CGTTTCTTCCAAAAAG
RT-PCR of *Clks*	EpClk_Sq	AGCGGAGGACTCATCCATGT	TCGTCTTGGGTTGCGTACTG	
Tissue distribution	Epper global	ATGGACGCACAATGAAAGGAGAAG	GGGCGCCGAAGTGTAACCA	
	Eptim global	GTGCGCCGAAATCCCTCAG	AGCCTTCTTCCCATCCAACTTCTT	
	EpClk global	TCTGCTGATGAAAGTTGTT	TATCGGCTACTTGCCCTTC	
	Epbal1 global	AACCTGATTGCCAACTGATTT	TGTTCGTGGTAGGGTGTG	
	Epcry2 global	CAAGAGATACCTTTCCCCT	ATCCTGGATGTGGCTGTCCT	
	Eprpl32 global	GAGGCGACTGTTCTTGAATAATGAG	CCAAAAGGTTCATTAGACAT	
Eurydice *period* dsRNAi	Epper dsRNAi	T7-GGACATGTTATTAGGACAATCCTTC	T7- TGAGCTGATTGAGCTTCCAG	
	Discoplax dsRNAi	T7- TCCTGCTTCCACGTACTCCT	T7- CCCTGAGGATGGTGACGAAG	
	**Primer name (Tm)**	**Forward sequence (5’→3’)**	**Reverse sequence (5’→3’)**	**Size of fragment (bp)**
Gene cloning	1st round Epper (42°C)	TGGGCTAYYTNCCNCARGA	CCGACNACRAAYTCNARYTT	237
	2nd round Epper (45°C)	ACTTCTAYCAYCMNGANGA	TTGYGGSACCANGGRTTNAC	177
	1st round Eptim (42°C)	GAYTGYGGITAYGGNACNCA	CCARTCRTCRCANADYTCR	1056
	2nd round Eptim (42°C)	AARRARTTYACNGTNGAYTT	GTRAANAYRCARTCRTTNAY	678
	1st round EpClk (40°C)	TGGAARTTYYTNTTYYTNGAYCA	CCADATCCAYTGYTGNCCYTT	198
	2nd round EpClk (45°C)	AYCAYMGNGCNCCNCCANT	TGNCCYTTNGTNARRAANCKRTA	168
	1st round Epbmal1 (42°C)	TCCGNATGGCNGTNCARCA	TGTGGAAYRTCNGTYTTNAC	363
	2nd round Epbmal1 (42°C)	TTCCTITTYGTNGTNGGNTG	GGCARCATNGTYTTNGCRTC	207
	1st round Epcry2 (42°C)	GARGARYTIGGNTTYGAYAC	ATRWARTCICCRTTNGGRTC	669
	2nd round Epcry2 (46°C)	GARMGIAARGCNTGGGTNGC	ACYTTCATNCCYTCYTCCCA	425
	1st round Epck1e (45°C)	GARGTNGCNATHAARYTHGA	GCNGCYTTNARNCCYTGCCA	555
	2nd round Epck1e (45°C)	AARCCNGAYAAYTTYYTNATG	TCDATNCCNARRTGNGTRTT	177

T7 seq: TAATACGACTCACTATAGGG

Accession numbers for protein sequences included in the phylogenetic analyses

	PERIOD	BMAL1/CYCLE	CLOCK	TIMELESS	TIMEOUT
Drosophila melanogaster	NP_525056.2	NP_524168.2	NP_001014576.1	P49021.2	NP_524341.3
Anopheles gambiae	-	XP_556301.3	XP_315720.4	XP_001689006.1	XP_001689129.1
Aedes aegypti	XP_001658976.1	-	-	XP_001657784.1	XP_001660177.1
Tribolium castaneum	NP_001106933.1	NP_001107795.1	NP_001106937.1	NP_001106934.1	EEZ99220.1
Apis mellifera	NP_001011596.1	XP_001121441.2	XP_394233.4	No	XP_003250653.1
Danaus plexippus	AAO48719.1	AAR13012.1	AAR13011.1	AAR15505.1	EHJ76705.1
Antheraea pernyi	Q17062.1	AAR14937.1	AAR14936.1	AAF66996.1	-
Bombyx mori	-	-	-	NP_001037622.1	BGIBMGA012558-TA[2]
Eurydice pulchra	AGV28714	AGV28715	AGV28720[3]	AGV28716	-
Daphnia pulex[1]	NCBI_GNO_212404	NCBI_GNO_1144014	NCBI_GNO_118274	NCBI_GNO_238244	NCBI_GNO_88254
Ixodes scapularis	XP_002410891.1	-	-	XP_002401472.1	-
Homo sapiens	-	BAA19935.1	AAF13733.1	-	BAA36499.1
Mus musculus	NP_035195.2 (PER1) /NP_035196.2 (PER2)	BAD26600.1	NP_031741.1	-	BAA76390.2
Danio rerio	NP_001025354.1 (PER1) /NP_878277.1 (PER2)	NP_571652.1	NP_571032.1	-	XP_003199460.1
Xenopus laevis	-	AAW80970.1	NP_001083854.2	-	BAE45344.1

	Drosophila-like CRYPTOCHROME	Mammalian-like CRYPTOCHROME	6-4 PHOTOLYASE
Drosophila melanogaster	NP_732407.1	No	NP_477188.1
Anopheles gambiae	XP_321104.4	ABB29887.1	XP_314748.3
Tribolium castaneum	No	NP_001076794.1	-
Apis mellifera	No	NP_001077099.1	-
Danaus plexippus	AAX58599.1	ABA62409.1	ABO38436.1
Antheraea pernyi	AAK11644.1	ABO38435.1	-
Bombyx mori	NP_001182628.1	NP_001182627.1	BGIBMGA000221-TA[2]
Eurydice pulchra	-	AGV28717	AGV28718
Euphausia superba	-	CAQ86665.1	-
Daphnia pulex	NCBI_GNO_454364	NCBI_GNO_88183	NCBI_GNO_1202103
Mus musculus	No	NP_031797.1 (CRY1) / NP_034093.1 (CRY2)	-
Danio rerio	No	NP_001070765.2 (CRY1a) / NP_571865.4 (CRY1b) NP_571866.1 (CRY2a) / NP_571867.1 (CRY2b)	NP_571863.1
Xenopus laevis	No	NP_001081129.1 (CRY1) NP_001082139.1 (CRY2a) /NP_001083936.1(CRY2b)	NP_001081421.1

[1] Accession numbers for all sequences of *Daphnia pulex* obtained from database 2007 (http://wfleabase.org) and updated to Genes 2010 (Beta3). [2] Acc no. *Bombyx mori* obtained from http://silkworm.genomics.org.cn/ silkdb. '-' not used or not identified. 'No' does not exist. [3] *Eurydice pulchra* clock isoform EpCLK5.

Supplemental References

1. Rubin, E.B., Shemesh, Y., Cohen, M., Elgavish, S., Robertson, H. M., and Bloch, G. (2006) Molecular and phylogenetic analyses reveal mammalian-like clockwork in the honey bee (*Apis mellifera*) and shed new light on the molecular evolution of the circadian clock. Genome Res. *16*, 1352-1356.

2. Saez L, Young MW. (1996). Regulation of nuclear entry of the Drosophila clock proteins period and timeless. Neuron 17, 911-920.

3. Wilcockson, D.C., Zhang, L., Hastings, M.H., Kyriacou, C.P., and Webster, S.G. (2011). A novel form of pigment-dispersing hormone in the central nervous system of the intertidal marine isopod, *Eurydice pulchra* (leach). J. Comp. Neurol. *519,* 562-575.

4. Allebrandt, K.V., Amin, N., Muller-Myhsok, B., Esko, T., Teder-Laving, M., Azevedo, R.V., Hayward, C., van Mill, J., Vogelzangs, N., Green, E.W*., et al.* (2013). A K(ATP) channel gene effect on sleep duration: from genome-wide association studies to function in Drosophila. Mol. Psychiatry *18,* 122-132.

5. Hogben, L., and Slome, D. (1931). The pigmentary effector system. VI. The dual character of endocrine coordination in amphibian colour change. Proc. Roy. Soc. B *108,* 10-53.

6. Larkin, M.A., Blackshields, G., Brown, N.P., Chenna, R., McGettigan, P.A., McWilliam, H., Valentin, F., Wallace, I.M., Wilm, A., Lopez, R*., et al.* (2007). Clustal W and Clustal X version 2.0. Bioinformatics *23,* 2947-2948.

7. Clamp, M., Cuff, J., Searle, S.M., and Barton, G.J. (2004). The Jalview Java alignment editor. Bioinformatics *20,* 426-427.

8. Tamura, K., Peterson, D., Peterson, N., Stecher, G., Nei, M., and Kumar, S. (2011). MEGA5: molecular evolutionary genetics analysis using maximum likelihood, evolutionary distance, and maximum parsimony methods. Mol. Biol. Evol. *28,* 2731-2739

9. Letunic, I., Doerks, T., and Bork, P. (2009). SMART 6: recent updates and new developments. Nucleic Acids Res. *37,* D229-32.

10. Marchler-Bauer, A., Anderson, J.B., Chitsaz, F., Derbyshire, M.K., DeWeese-Scott, C., Fong, J.H., Geer, L.Y., Geer, R.C., Gonzales, N.R., Gwadz, M*., et al.* (2009). CDD: specific functional annotation with the Conserved Domain Database. Nucleic Acids Res. *37,* D205-10.

11. Nguyen Ba, A.N., Pogoutse, A., Provart, N. and Moses, A,M. (2009) NLStradamus: a simple Hidden Markov Model for nuclear localization signal prediction. BMC Bioinformatics. *10,* 202.

12. Kosugi, S., Hasebe, M., Tomita, M. and Yanagawa, H. (2009) Systematic identification of cell cycle-dependent yeast nucleocytoplasmic shuttling proteins by prediction of composite motifs. Proc Natl Acad Sci U S A. *106,* 10171-6.

13. la Cour, T., Kiemer, L., Molgaard, A., Gupta, R., Skriver, K., and Brunak, S. (2004). Analysis and prediction of leucine-rich nuclear export signals. Protein Eng. Des. Sel. *17,* 527-536.

14. Darlington, T.K., Wager-Smith, K., Ceriani, M.F., Staknis, D., Gekakis, N., Steeves, T.D., Weitz, C.J., Takahashi, J.S., and Kay, S.A. (1998). Closing the circadian loop: CLOCK-induced transcription of its own inhibitors per and tim. Science *280,* 1599-1603.

15. Emery, P., So, W.V., Kaneko, M., Hall, J.C., and Rosbash, M. (1998). CRY, a Drosophila clock and light-regulated cryptochrome, is a major contributor to circadian rhythm resetting and photosensitivity. Cell *95,* 669-679.

16. Yang, Z., and Sehgal, A. (2001). Role of molecular oscillations in generating behavioral rhythms in Drosophila. Neuron *29,* 453-467.

17. Brand, A.H., and Perrimon, N. (1993). Targeted Gene-Expression as a Means of Altering Cell Fates and Generating Dominant Phenotypes. Development *118,* 401-415.

18. Rosato, E., and Kyriacou, C.P. (2006). Analysis of locomotor activity rhythms in Drosophila. Nat. Protoc. *1,* 559-568.

19. Sternberger, L.A. (1979). Immunocytochemistry (New York: Wiley Medical).
